# Characterization of a glycan-binding complex of minor pilins completes the analysis of *Streptococcus sanguinis* type 4 pili subunits

**DOI:** 10.1073/pnas.2216237120

**Published:** 2023-01-10

**Authors:** Meriam Shahin, Devon Sheppard, Claire Raynaud, Jamie-Lee Berry, Ishwori Gurung, Lisete M. Silva, Ten Feizi, Yan Liu, Vladimir Pelicic

**Affiliations:** ^a^Medical Research Council Centre for Molecular Bacteriology and Infection, Imperial College London, SW7 2AZ London, United Kingdom; ^b^Glycosciences Laboratory, Department of Metabolism, Digestion and Reproduction, Imperial College London, W12 0NN London, United Kingdom; ^c^Laboratoire de Chimie Bactérienne, CNRS/Aix-Marseille Université (Unité Mixte de Recherche 7283), Institut de Microbiologie de la Méditerranée, 13009 Marseille, France

**Keywords:** type 4 pili, pilin, adhesion

## Abstract

Type 4 filaments (T4F)—of which type 4 pili (T4P) are the archetype—are a superfamily of nanomachines nearly ubiquitous in prokaryotes. T4F are polymers of one major pilin, which also contain minor pilins whose roles are often poorly understood. Here, we complete the structure/function analysis of the full set of T4P pilins in the opportunistic bacterial pathogen *Streptococcus sanguinis*. We determined the structure of the minor pilin PilA, which is unexpectedly similar to one of the subunits of a tip-located complex of four minor pilins, widely conserved in T4F. We found that PilA interacts and dramatically stabilizes the minor pilin PilC. We determined the structure of PilC, showing that it is a modular pilin with a lectin module binding a subset of glycans prevalent in the human glycome, the host of *S. sanguinis*. Altogether, our findings support a model whereby the minor pilins in *S. sanguinis* T4P form a tip-located complex promoting adhesion to various host receptors. This has general implications for T4F.

## Significance

Type 4 pili (T4P)—important in bacterial pathogens—are filaments composed of one well-characterized major pilin, and several minor pilins whose roles are often poorly understood. *Streptococcus sanguinis* T4P are composed of five pilins, which makes it a good model to determine the role of each pilin subunit in detail. Here, we characterize PilA and PilC, showing how they interact and how they function. Together with our previous findings, this provides an integrated view of the role of the five pilin subunits in *S. sanguinis* T4P. PilE1/PilE2 are the major pilins forming the backbone of the filament, while the three minor pilins (PilA, PilB, and PilC) form a tip-located complex promoting adhesion to various host receptors.

Type 4 filaments (T4F)—type 4 pili (T4P) and type 2 secretion systems (T2SS) being the best known—are a superfamily of nanomachines ubiquitous in prokaryotes (1, 2). T4F, which mediate a wide variety of functions (1), are composed of type 4 pilins, assembled by conserved multiprotein machineries spanning the cell envelope (3). T4F have been studied for decades because they are virulence factors in many human pathogens.

Our current understanding of T4F is as follows. T4F are polymers of type 4 pilins: one predominant (major) and several low abundance (minor) subunits (4). These subunits are synthesized as prepilins with a distinctive N-terminal sequence motif—the class 3 signal peptide (SP) (5)—consisting of a short hydrophilic leader peptide ending with a Gly or Ala, followed by a hydrophobic tract of 21 residues, which anchors prepilins in the cytoplasmic membrane (CM). Pilins display a characteristic "lollipop" structure where the hydrophobic tract of the SP constitutes the N-terminal portion (α1N) of an α-helix of approx. 50 residues (α1) that supports a globular head (4). However, larger minor pilins—displaying extra domains "grafted" onto a pilin moiety and hence defined as modular (6)—are not uncommon, which partly explains the extreme functional versatility of T4F. Pilins are assembled in filaments at the CM (3) in two steps. The leader peptide in prepilins is first cleaved by a dedicated prepilin peptidase (PPase) (7–9), before the pilins are polymerized by a machinery centered on a platform protein and a hexameric ATPase (10). All T4F are helical polymers where pilins are held together by extensive interactions between their α1-helices, which pack within the core of the filament, with often a portion of α1N “melted” (11). However, different helical symmetry parameters are seen in different T4F (12–16).

While the role of major pilins is clear, i.e., they constitute the backbone of T4F, the role and precise localization of the minor pilins remain poorly defined (4). Typically, minor pilins are divided into two categories: widely conserved and nonconserved (or system-specific) (4). Nonconserved minor pilins are highly variable, and their roles cannot be predicted. In some T4F, these pilins have been well studied, such as for example ComP, PilV, PilX in *Neisseria meningitidis* T4P. These three pilins are dispensable for filament assembly but are key for different T4P-mediated functions. ComP binds DNA to promote its uptake during natural transformation (17, 18), PilV promotes bacterial adhesion to human cells (19), and PilX supports the formation of bacterial aggregates (20). In contrast, a group of four minor pilins—often labeled with the letters H, I, J, K—are widely conserved in different T4F and essential for filament assembly (21–23). Their role and mechanism of action are likely to be the same since HIJK sets from different T4F are functionally interchangeable in promoting filament assembly (24). These pilins interact to form a quasihelical complex (25, 26), which is capped by the bulky K subunit. This suggested a localization at the pilus tip (25, 26), which was recently confirmed by cryoelectron tomography (cryo-ET) of *Myxococcus xanthus* T4P (27). Since T4F are assembled from tip to base (3), the HIJK complex is therefore thought to initiate filament assembly (28, 29).

Recently, the study of T4F in monoderm bacteria opened new research avenues (30), with *Streptococcus sanguinis* becoming a cutting-edge model (31). This commensal of the human oral cavity (32) is an opportunistic pathogen frequently causing infective endocarditis (IE) (33). It uses an elementary machinery to assemble T4P composed of two major (PilE1 and PilE2) and three minor (PilA, PilB, and PilC) pilins (34, 35). This makes it possible to characterize the role of all pilin subunits in *S. sanguinis* T4P in detail. Since we previously characterized PilE1, PilE2, and PilB (6, 34, 35), here we focused on PilA and PilC. We report the detailed structure/function analysis of these two minor pilins, showing how they interact, and how they function. This provides an integrated view of the role of all the pilin subunits in a defined T4F, which has implications for this widespread superfamily of nanomachines.

## Results

### PilA and PilC Minor Pilins have Remarkably Different Characteristics.

**Fig. 1.**
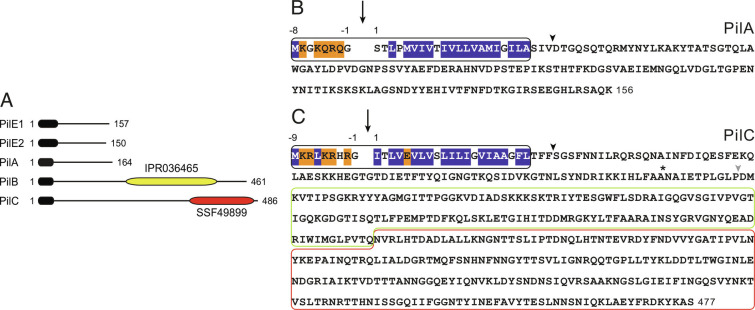
PilA and PilC sequence characteristics. (*A*) Protein architecture of the five prepilins in the T4P-encoding *pil* locus in *S. sanguinis* 2908 (34). Each protein harbors a type 4 pilin-defining class 3 SP (black rounded rectangle) at its N terminus. PilB and PilC are modular pilins, which contain additional domains at their C terminus (yellow and red rounded rectangles). PilB contains a von Willebrand factor A-like domain (IPR036465) (6), while PilC contains a concanavalin A-like lectin/glucanase structural domain (SSF49899). Proteins are drawn to scale. (*B*) Relevant sequence features of PilA. The class 3 SP is black boxed and contains an 8-aa long leader peptide with mostly hydrophilic (orange shading) and neutral (no shading) residues, followed by a tract of 21 mainly hydrophobic residues (blue shading). The processing of the leader peptide by the PPase, indicated by the vertical arrow, generates a pilin of 17 kDa. The arrowhead indicates the soluble portion of PilA that was produced and purified in 6His–PilA. (*C*) Relevant sequence features of PilC. The class 3 SP is black boxed. The processing of the leader peptide by the PPase (black vertical arrow), generates a pilin of 52.8 kDa. The lectin module (SSF49899) is red boxed. The Ig-like fold module revealed by our structure is green boxed. Arrowheads indicate the soluble proteins that were produced and purified in this study: black arrowhead is 6His–PilC, gray arrowhead is 6His–PilC_Δpilin_ without the pilin moiety. The site of spontaneous proteolysis in 6His–PilC is indicated by a star.

Previously, we showed that all the genes involved in T4P biology in *S. sanguinis* 2908 strain cluster together in a 22-kb locus named *pil* (34). This locus encodes five pilins—PilE1, PilE2, PilA, PilB, and PilC (Fig. 1*A*)—which are essential for piliation (34). These proteins are processed by the PPase PilD and become subunits of T4P (34, 35). PilE1 and PilE2 are the major pilins, whereas PilA, PilB, and PilC are minor pilins (35). We previously characterized PilE1, PilE2, and PilB (6, 35). Here, we complete the analysis of the role of pilins in *S. sanguinis* T4P by focusing on PilA and PilC.

Although it has a typical pilin size—17 kDa for the processed protein—PilA is an unusual pilin. Its class 3 SP, which was identified by visual inspection (34), is degenerate (Fig. 1*B*) and could not be identified by any of the available bioinformatic tools, including PilFind dedicated to this purpose (36). This is mainly due to the absence of the typical Glu residue in the fifth position of the processed protein (Glu_5_) (23) and the presence of an uncommon Pro_4_ (Fig. 1*B*). PilA has no sequence homologs outside of *S. sanguinis*, which makes it impossible to predict its role.

Compared with PilA, PilC has radically different features. It has a canonical class 3 SP (Fig. 1*C*), with a typical Glu_5_ in the processed protein. Processed PilC is unusually large for a pilin, with a theoretical molecular mass of 52.8 kDa. As recently reported for PilB (6), the large size of PilC is due to its predicted modular architecture, with a domain grafted onto what appears to be an unusually short pilin moiety (Fig. 1*C*). In PilC, this extra module, readily identifiable by sequence-based bioinformatics, belongs to the concanavalin A-like lectin/glucanase domain superfamily (SSF49899), which is composed of carbohydrate-binding proteins in the three domains of life with different ligand specificities. These observations raise the possibility that PilC is a modular pilin with a lectin module promoting T4P-mediated adhesion of *S. sanguinis* to glycans.

### PilA Is Structurally Similar to a Widely Conserved Subunit from a Complex of Minor Pilins Found at the Tip of Many T4F.

**Fig. 2.**
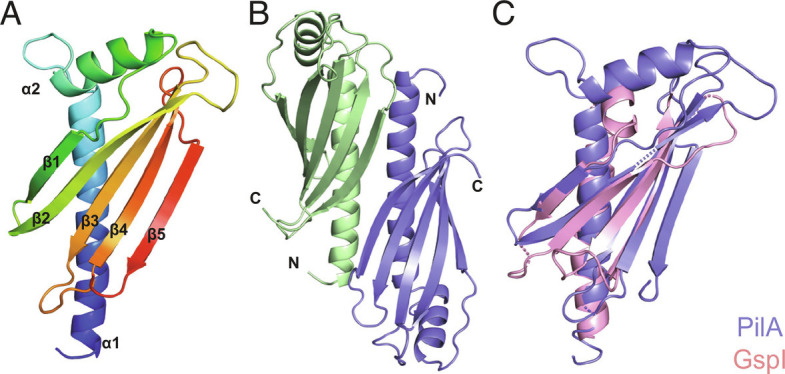
Crystal structure of PilA. (*A*) Cartoon view of the PilA structure rainbow-colored from blue (N terminus) to red (C terminus). (*B*) Cartoon view of the head-to-toe PilA dimers in the crystal packing. (*C*) Structural similarity between PilA (blue) and a widely conserved minor pilin in T4F (pink). The GspI protein from enterotoxigenic *E. coli* T2SS (PDB 3CI0) is presented. Although these two proteins share only 9.4% sequence identity, they superpose with an rmsd of 1.52 Å.

To better understand the role of PilA, we solved its 3D structure by X-ray crystallography. We produced a 15.7-kDa recombinant protein in *Escherichia coli*, in which the N-terminal 24 residues of the mature pilin (Fig. 1*B*) were replaced by a hexahistidine tag (6His). This usually promotes pilin solubility without structurally impacting the rest of the protein (37). We purified soluble 6His–PilA using a combination of affinity and size-exclusion chromatography (SEC), noticing that it purifies as a dimer. The protein crystallized readily. After optimizing the best diffracting crystals and using protein produced in the presence of seleno-methionine (SeMet) for phase determination, we solved a 1.77-Å resolution structure (*SI Appendix*, Table S1). As can be seen in Fig. 2*A*, PilA—which looks like an “ice axe”—exhibits a typical pilin architecture, with an N-terminal α1-helix packed against a β-sheet composed of five antiparallel β-strands. Modeling the full-length PilA protein using AlphaFold (38) reveals a canonical lollipop shape (4) (*SI Appendix*, Fig. S1). The almost perfect superposition of our structure with the AlphaFold prediction (*SI Appendix*, Fig. S1)—with a mere 0.58 Å rmsd—validates the accuracy of both AlphaFold (38) and our structure.

A closer inspection of the crystal packing showed that PilA forms head-to-toe dimers (Fig. 2*B*), stabilized by a series of hydrogen bonds between residues in α1-helix and the last two β-strands of the β-sheet (β4 and β5) (*SI Appendix*, Fig. S2). This explains why 6His–PilA purifies as a dimer. Strikingly, while PilA exhibits no sequence homology to other pilins, our structure reveals extensive structural similarity to the I subunit of HIJK tip-located complex of conserved minor pilins (25, 26) in a variety of T4F. For example, PilA superposes with an rmsd of 1.52 Å with GspI (PDB 3CI0) from enterotoxigenic *E. coli* T2SS (Fig. 2*C*). Critically, since I interacts with J and K (25, 26), this suggested to us that PilA might be interacting with another minor pilin (PilB and/or PilC) to form a complex capping *S. sanguinis* T4P.

### PilA Interacts with PilC.

**Fig. 3.**
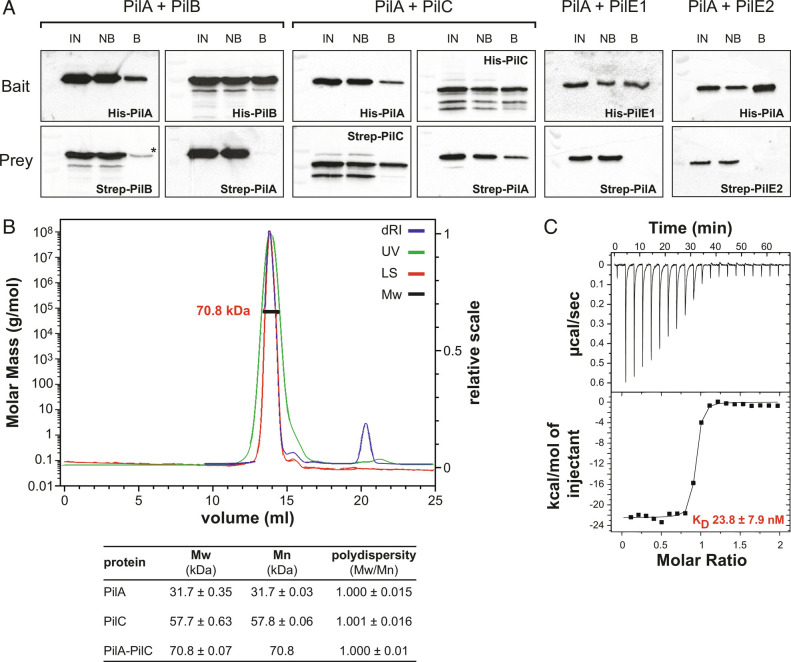
PilA interacts with PilC. (*A*) Interactions between PilA and the four other pilins were tested by performing pull-down assays using purified proteins corresponding to the soluble portion of the pilins. The 6His-tagged bait protein was first incubated with Strep-tagged prey protein, and then pulled-down using cobalt-based magnetic beads. Proteins bound to the beads were eluted and identified by immunoblotting using specific anti-Pil antibodies. IN, input. NB, not bound. B, bound. *Nonspecific binding of Strep-PilB to the beads, which was also observed with the protein on its own. (*B*) Characterization of the interaction between PilA and PilC by SEC–MALS. (*Upper*) chromatogram traces for the PilAC complex. dRI, differential refractive index, LS, MALS signal. Mw, weight average molecular weight. (*Lower*) SEC–MALS data for PilA, PilC and PilA–PilC. Mn, number average molecular weight. (*C*) Quantification of the PilA–PilC interaction by ITC. A representative ITC titration curve (*Upper*) and binding isotherm (*Lower*) are presented. The calculated K_D_ is the average ± SD from three independent experiments.

To identify interactions between PilA and other pilins in *S. sanguinis* T4P, we performed pull-down assays with proteins purified using two different affinity tags: 6His and Strep-tag II. In brief, i) purified proteins corresponding to the soluble portion of the pilins were mixed in solution, ii) the 6His-tagged bait was selectively captured using cobalt-based magnetic beads, and iii) pull-down of the Strep-tagged prey was assessed by immunoblotting using specific anti-Pil antibodies. We verified beforehand that Strep-tagged proteins were not captured using these beads (although PilB exhibited very limited binding). Interestingly, while the bait proteins were always captured, prey proteins were pulled down only for the PilA/PilC pair (Fig. 3*A*), both by 6His–PilA and 6His–PilC.

To characterize the PilAC complex, we used SEC coupled with multiangle light scattering (SEC–MALS), which allows for absolute characterization of molecular mass and stoichiometry of proteins in solution. SEC–MALS was first used with 6His–PilA and 6His–PilC separately. Both proteins eluted as single, monodisperse peaks with estimated molecular masses (in kDa) of 31.7 ± 0.35, and 57.7 ± 0.63, respectively (Fig. 3*B*). For 6His–PilA, this value is significantly higher than the theoretical molecular mass (15.7 kDa), confirming that the purified protein is a dimer in solution. When SEC–MALS was performed after mixing equimolar amounts of purified 6His–PilA and 6His–PilC, we observed a single, monodisperse peak of 70.8 ± 0.07 kDa (Fig. 3*B*), indicating that the PilAC complex has a 1:1 stoichiometry. The disappearance of the PilA–PilA homodimer shows that PilC is the preferred binding partner of PilA. Using isothermal titration calorimetry (ITC), we showed that PilA has high affinity for PilC (Fig. 3*C*), with a dissociation constant (K_D_) of 23.8 ± 7.9 nM.

**Fig. 4.**
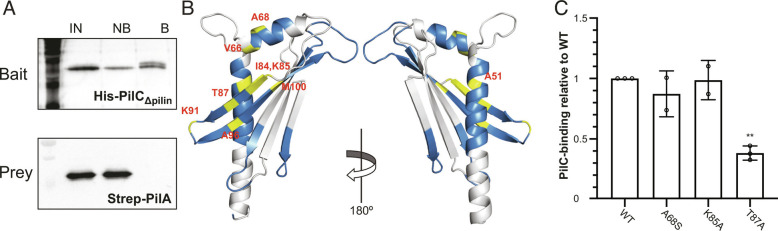
Mapping the PilA–PilC binding interface. (*A*) Testing the interaction between PilA and PilC_Δpilin_ by performing pull-down assays. PilC_Δpilin_ corresponds to the protein missing the pilin moiety. Proteins bound to the beads were eluted and identified by immunoblotting using anti-6His and anti-PilA antibodies. IN, input. NB, not bound. B, bound. (*B*) NMR analysis of PilAC complex formation. PilA residues for which NMR assignment could be performed are highlighted in blue on our crystal structure (180° view). Residues experiencing significant chemical shift perturbations in the presence of PilC are highlighted in yellow. (*C*) ITC quantification of the binding of PilA mutant proteins to PilC. The results, presented as K_D_ ratios to the WT, are the average ± SD from 2 to 3 independent experiments. Statistical significance was calculated using one-way ANOVA followed by Dunnett’s multiple comparison tests.

We then assessed how PilA and PilC interact. We first performed pull-down assays using a purified 6His–PilC_Δpilin_ protein lacking the pilin moiety (Fig. 1*C*). In contrast to 6His–PilC (Fig. 3*B*), 6His–PilC_Δpilin_ was unable to pull down its Strep-PilA partner (Fig. 4*A*). This suggests that PilA interacts with the pilin moiety of PilC. Next, we used multidimensional NMR to identify the portion of PilA binding to PilC. First, we performed a partial NMR assignment of the resonances in 6His–PilA (*SI Appendix*, Fig. S3), highlighting the assigned residues on our crystal structure (Fig. 4*B*). Then, we identified the chemical shift perturbations occurring in PilA when PilC was added (*SI Appendix*, Fig. S3). Nine chemical shifts perturbations concerned PilA residues not involved in the formation of PilA dimers, which are therefore likely to be involved in the PilA-PilC interaction (Fig. 4*B*). The PilAC interaction interface involves the α1 and α2 helices and the first 2 β-strands (β1 and β2) of PilA (Fig. 4*B*). Curiously, although this interface is distinct from the one involved in the formation of PilA homodimers (*SI Appendix*, Fig. S2), the formation of a PilAC complex led to the disappearance of the PilA dimer. We probed this interaction interface by mutagenesis of Ala_68_, Lys_85_ and Thr_87_ in PilA, and quantification of the ability of these mutant proteins to interact with PilC using ITC (Fig. 4*C*). This showed that PilA_T87A_ displayed a significantly reduced affinity for PilC, corresponding to 38.3 ± 5.9% of the wild-type (WT) binding.

Taken together, these experiments show that PilA and PilC interact, confirming our hypothesis that PilA is part of a complex of minor pilins in *S. sanguinis* T4P.

### PilA Stabilizes PilC.

**Fig. 5.**
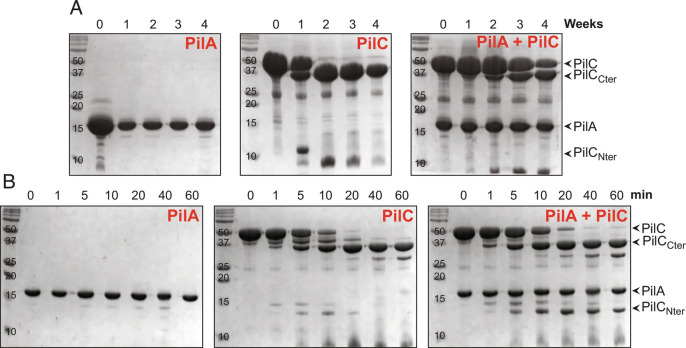
PilA stabilizes PilC. (*A*) Long-term protein stability experiments. Purified PilA and PilC were mixed in equimolar amounts, and the PilAC complex was isolated by SEC. The complex, as well as PilA and PilC on their own, were kept at 4 °C for a month. Samples were taken once a week and analyzed by SDS–PAGE/Coomassie. The degradation products (PilC_Nter_ and PilC_Cter_) are identified on the right side. Three independent experiments were performed yielding same results, representative gels are shown. The molecular masses are in kDa. (*B*) Trypsin sensitivity assays. Purified PilA, PilC, and PilAC were incubated with trypsin (1:1,000 dilution) for 60 min on ice. Samples were taken at various times and analyzed by SDS–PAGE/Coomassie. The degradation products are indicated on the right side. Three independent experiments were performed yielding similar results, representative gels are shown.

We noticed that purified 6His–PilC was unstable, which prompted us to perform long-term stability tests. We kept the purified protein at 4 °C, took samples once a week, which we analyzed by SDS–PAGE/Coomassie. This revealed that 6His–PilC was completely degraded by week two (Fig. 5*A*), yielding two degradation products. Edman sequencing showed that proteolysis occurred after Ala_101_ close to the end of the pilin moiety (Fig. 1*C*), hence the two degradation products were named PilC_Nter_ (smaller N-terminal product) and PilC_Cter_ (larger C-terminal product). PilC_Nter_ is highly unstable as it was no longer detectable by week two. In the same conditions, 6His–PilA remained unchanged over 4 wk, confirming that it is a very stable protein (Fig. 5*A*). Interestingly, when the PilAC complex was analyzed in the same way, PilC degradation was significantly delayed. In the presence of PilA, PilC degradation began only at week three, and even at week four the majority of PilC was still intact (Fig. 5*A*).

The stabilizing effect of PilA on PilC was further characterized by performing trypsin sensitivity assays. In brief, we incubated proteins with trypsin on ice for 60 min, took samples at specific timepoints, which we analyzed by SDS–PAGE/Coomassie (Fig. 5*B*). As above, 6His–PilA was intact over the course of the assay, while 6His–PilC the protein was entirely converted into PilC_Nter_ and PilC_Cter_ by 20 min. As above, the pilin moiety PilC_Nter_ was unstable and no longer detectable after 40 min. In contrast, when it was part of a complex with 6His–PilA, 6His–PilC degradation was delayed (Fig. 5*B*). In addition, PilC_Nter_ was also stabilized as it was still detectable after 60 min.

Taken together, these findings show that the functional consequence of the PilAC interaction is that PilA stabilizes PilC, especially its pilin moiety.

### Crystal Structures of PilC Reveal a Modular Pilin.

**Fig. 6.**
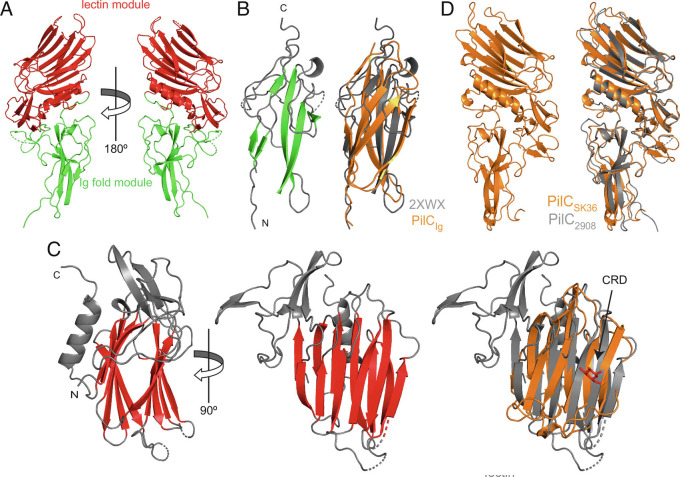
Crystal structures of PilC. (*A*) Cartoon views at 180° rotation of the structure of PilC_Δpilin_ from strain 2908 in which two distinct modules have been identified highlighted in green (Ig-like fold) and red (lectin). (*B*, *Left*) Close-up view of the Ig-like fold module (PilC_Ig_). The two β-sheets packed against each other have been highlighted in green. Right, superposition of PilC_Ig_ with the Ig-like fold domain in *V. cholerae* colonization factor GbpA (PDB 2XWX) (40). (*C*, *Left*) orthogonal cartoon views of the lectin module (PilC_lectin_) in which the β-sandwich of two opposing antiparallel β-sheets is highlighted in red. The putative CRD is indicated by an arrow. (*C*, *Right*) superposition of PilC_lectin_ with human Gal-7 (PDB 2 GAL) (41). The bound galactose in the Gal-7 structure is highlighted in red. (*D*, *Left*) structure of PilC from *S. sanguinis* SK36. Right, superposition of PilC^2908^ and PilC^SK36^. These two proteins, which share 57% sequence identity (*SI Appendix*, Fig. S4), superpose almost perfectly with an rmsd of only 0.79 Å.

To confirm the prediction that PilC might be a modular pilin, we solved its 3D structure by X-ray crystallography. Due to the instability of its pilin moiety, we produced a 41.4-kDa recombinant 6His–PilC__Δpilin__ protein in *E. coli* lacking the pilin moiety (Fig. 1*C*). This protein crystallized readily. After optimizing the best diffracting crystals and using SeMet protein for phase determination, we solved a high-resolution structure at 1.45 Å (*SI Appendix*, Table S1). Strikingly, while bioinformatics only predicted that the C terminus of PilC corresponds to a lectin domain, our structure reveals two distinct modules (Fig. 6*A*). The first module adopts an Ig-like fold (PilC_Ig_), consisting of two β-sheets packed against each other (Fig. 6*B*). This is a widespread domain found in proteins of different functions (39), which is thought to be involved in protein-protein interactions. PilC_Ig_ thus shows significant structural similarity to multiple structures, including the N-acetylglucosamine-binding protein GbpA from *Vibrio cholerae* (40) (PDB 2XWX). The Ig-like domains in these two proteins, which display no sequence homology, superpose with an rmsd of 2.17 Å (Fig. 6*B*).

The second module that corresponds to the lectin (PilC_lectin_), is a β-sandwich characterized by two opposing antiparallel β-sheets of six strands (Fig. 6*C*), with concave and convex sides. PilC_lectin_ shows significant structural similarity to multiple carbohydrate-binding proteins, including galectins such as human Gal-7 (PDB 2 GAL) (41). PilC_lectin_ and Gal-7, which display no sequence homology, superpose with an rmsd of 1.95 Å (Fig. 6*C*). In the Gal-7 structure (Fig. 6*C*), the protein interacts with galactose via a carbohydrate-recognition domain (CRD) forming a patch on the concave side of the protein (41). Since the two proteins share good structural homology in that region (Fig. 6*C*), we speculate that the CRD might be on the concave side of PilC_lectin_.

To try to determine a PilC structure encompassing the pilin moiety, we expressed and crystallized PilC from another *S. sanguinis* strain SK36 (PilC^SK36^), in which the N-terminal 24 residues of the processed protein were replaced by 6His. After collecting a complete dataset, we solved a 1.60 Å resolution structure (*SI Appendix*, Table S1). Surprisingly, although the purified protein contained the pilin moiety, this portion was missing in the crystals. It must therefore have been degraded during prolonged incubation in the crystallization trays due to its inherent instability. When we compared our two PilC structures, we found that they are essentially identical, superposing onto each other with an rmsd of only 0.79 Å (Fig. 6*D*). This suggests that these proteins—showing 57% sequence identity (*SI Appendix*, Fig. S4)—are likely to bind similar glycan ligands. Finally, we used AlphaFold to model the full-length PilC, revealing a lollipop with a bulky domain grafted (*SI Appendix*, Fig. S5*A*), which superposes nicely onto our crystal structure (1.03 Å rmsd). Interestingly, the globular head of PilC is strikingly different from known pilins. Instead of the usual β-sheet with several antiparallel β-strands, PilC displays a highly distorted β-sheet composed of three β-strands, where β1 and β3 are orthogonal to each other, and linked by β2 with a 90º kink (*SI Appendix*, Fig. S5*B*).

### PilC Binds Two Types of Glycans Prevalent in the Human Glycome.

**Fig. 7.**
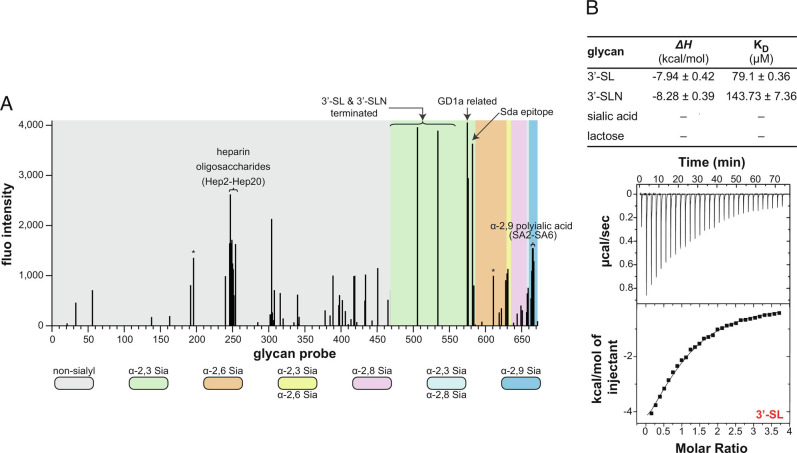
PilC specifically binds sialylated glycans and GAG. (*A*) Glycan microarray screening analyses of 6His–PilC, as a protein-antibody precomplex. Multiple experiments were performed yielding similar results. Results of one representative experiment are shown as the means of fluorescence intensities of duplicate spots of glycan probes, printed at 5 fmol/spot. The 672 lipid-linked probes are grouped according to sialyl linkages as annotated by the coloured panels. The full list of glycan probes, their sequences, and binding scores are given in Dataset S1. *Signals with large error bars due to artifacts on the array slides. (*B*) ITC quantification of PilC_Δpilin_ affinity for 3'-SL, 3'-SLN, sialic acid, and lactose. The calculated K_D_ are the average ± SD from three independent experiments. For binding to 3′-SL, representative ITC titration curve (*Upper*) and binding isotherm (*Lower*) are presented.

The presence of a lectin domain suggests that PilC might be an adhesin recognizing host glycans. We therefore investigated the glycan binding ability and specificity of PilC using a glycan microarray designed using the neoglycolipid (NGL) technology (42). 6His–PilC bound to a range of glycan probes—results are shown for 672 sequence-defined oligosaccharide probes (Dataset S1)—corroborating its lectin activity (Fig. 7*A*). Two major types of negatively charged glycans were bound: sialylated glycans and sulfated glycosaminoglycans (GAG). Most of the sialylated ligands display a α2-3-sialyl linkage and terminate with 3′-sialyllactose (3′-SL) or 3′-sialyl-N-acetyllactosamine (3′-SLN). Also well recognized (Fig. 7*A*) were ganglioside GD1a and four probes terminating with the Sd^a^ antigen, which is known to be present in human secretions including saliva (43, 44). A few probes displaying other sialyl linkages—including α2-9-polysialic acid—were also bound (Fig. 7*A*). The sulfated GAG ligands are mainly oligosaccharide probes derived from heparin, representative of the highly sulfated domains of heparan sulfate, which is ubiquitously expressed on the cell surface and in the extracellular matrix. Both sialylated glycans and GAGs are prevalent in the human glycome (45). We observed a similar binding profile for PilC^SK36^ (*SI Appendix*, Fig. S6), showing that glycan-binding ability is conserved in PilC orthologs.

We tested whether the lectin activity of PilC is affected by its pilin moiety, by probing the glycan microarray with 6His–PilC_Δpilin_. PilC_Δpilin_ bound the same type of glycans as PilC, suggesting that glycan binding is largely due to the lectin module. However, the pilin moiety affects the binding (*SI Appendix*, Fig. S7). When compared with PilC, PilC_Δpilin_ binds strongly to heparin oligosaccharide probes and weakly to a more restricted range of α2-3-linked sialyl glycans (*SI Appendix*, Fig. S7). Next, using ITC, we quantified the affinity of PilC_Δpilin_ for 3′-SL, 3′-SLN and their constituent subunits (sialic acid and lactose) (Fig. 7*B*). While no binding was measured with lactose or sialic acid, PilC bound both 3′-SL and 3′-SLN in accord with the microarray findings. There was a slight preference for 3′-SL over 3′-SLN, with K_D_ of 79.1 ± 0.36 µM and 143.73 ± 7.36 µM, respectively (Fig. 7*B*).

**Fig. 8.**
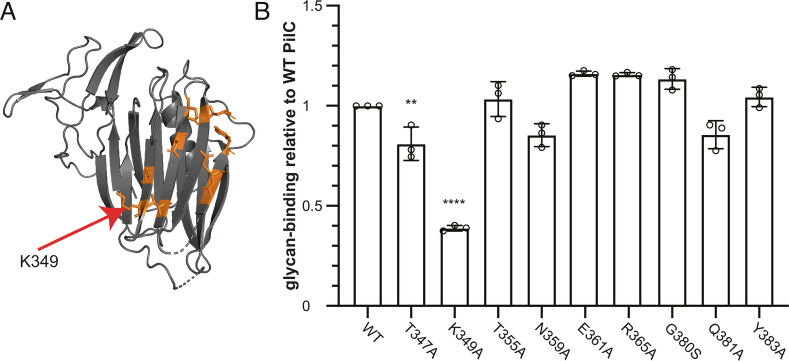
Probing the putative carbohydrate-recognition domain in PilC by mutagenesis. (*A*) Cartoon view of PilC_lectin_ where the residues in the putative CRD—targeted by SDM—are highlighted in orange. (*B*) ITC quantification of binding to 3′-SL by PilC_Δpilin_ mutant proteins. The results, presented as K_D_ ratios to the WT, are the average ± SD from three independent experiments. Statistical significance was calculated using one-way ANOVA followed by Dunnett’s multiple comparison tests.

Finally, we probed the glycan-binding activity of PilC by mutagenesis of residues on the concave side of PilC_lectin_ where the CRD is predicted to be (Fig. 6*C*). We made a series of PilC_Δpilin_ derivatives in which surface-exposed residues in the putative CRD were changed by site-directed mutagenesis (Fig. 8*A*). We then purified these mutant proteins and quantified their affinity for 3′-SL using ITC, before comparing it with WT PilC (*SI Appendix*, Fig. S8*B*). This revealed that PilC_T347A_ and PilC_K349A_ displayed significantly reduced affinity for 3′-SL, which was particularly pronounced for PilC_K349A_, with 39.3 ± 1.4% of the WT binding. This finding indicates that the concave side of its lectin module is involved in the ability of PilC to bind specific glycans.

### Modeling Predicts that the PilAC Complex Is Located at the Tip of *S. sanguinis* T4P, together with PilB.

**Fig. 9.**
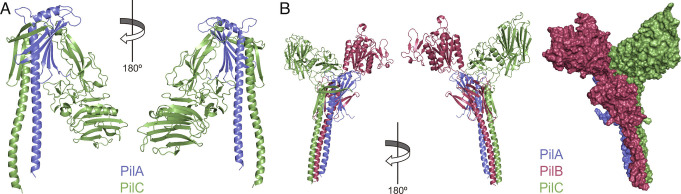
Modeling the complex of minor pilins in *S. sanguinis* T4P. (*A*) Model of the PilAC complex. (*B*) Model of the PilABC complex. *Left*, cartoon views at 180° rotation. *Right*, surface representation.

Since the instability of PilC pilin moiety precluded us from determining a crystal structure of the PilAC complex, we modeled this complex using AlphaFold (38). In the PilAC complex (Fig. 9*A*), the two proteins are staggered, PilA is on the top and the extra modules in PilC are on the side. PilA extends the β-sheet of PilC (*SI Appendix*, Fig. S8), which is likely to explain its stabilizsing effect on PilC. The complex is stabilized by a series of 14 hydrogen bonds and five salt bridges (*SI Appendix*, Fig. S8). The predicted PilAC interaction interface is in strikingly good agreement with that identified by NMR (Fig. 4*B*), including the α1 and α2 helices, and the β1 and β2 strands of PilA. This significantly strengthens the validity of our model.

*S. sanguinis* T4P contain another modular pilin, PilB, which was previously predicted to be at the pilus tip (6). We therefore wondered if PilA, B, C could coexist in one pilus, which we assessed by modeling using AlphaFold. The PilABC model suggests that the two modular pilins PilB and PilC can be accommodated in the same complex despite their bulky extra domains (Fig. 9*B*). The three subunits form a quasihelical complex in which PilA interacts with PilC, which interacts with PilB. Some level of movement for the extra modules in PilB and PilC is expected because the short loops that connect them to their respective pilin moieties are likely to be flexible. Although PilA is predicted to be added first, the extra modules in PilB and PilC are capping the complex and look like "open wings" (Fig. 9*B*). Due to this peculiar architecture, the PilABC complex could only be accommodated at the tip of the T4P. It is possible that a portion of α1N needs to be melted, as in major pilins in many T4F (11), to allow proper fitting. Tip localization is likely to optimize the presentation of the adhesive modules in PilB and PilC, promoting *S. sanguinis* adhesion to host surfaces.

## Discussion

T4F are a superfamily of filamentous nanomachines ubiquitous in bacteria and archaea (1, 2), mediating a wide variety of functions ranging from adhesion to protein secretion. Although T4F have been studied for 40 y—notably T4P (46)—many aspects of their complex biology remain poorly understood. This holds especially true for the filament—the "business end" of T4F nanomachines—since the roles of all pilin subunits are yet to be understood. Therefore, in this report we used the inherent simplicity of T4P in monoderm bacteria—which recently opened new research avenues (30)—to complete a structure/function analysis of all the pilins in *S. sanguinis* (31) T4P. This led to the notable findings discussed below, shining light on T4F biology.

Our first important finding—with widespread implications in T4F biology—is the unexpected discovery that the minor pilin PilA from *S. sanguinis* is a structural homolog of the I minor pilin of the widely conserved HIJK complex. The broader than predicted conservation of that minor pilin is a testament to the central role it plays in T4F biology. There are multiple analogies between the two systems, suggesting that PilABC is perhaps a rudimentary/ancestral version of HIJK. The H,I,J,K pilins—which are found in different T4F and are functionally interchangeable (24)—are encoded by an operon, and K lacks the usually conserved Glu_5_ (4). PilA,B,C pilins are also encoded by an operon, and PilA lacks Glu_5_ (35). Structural studies showed that HIJK interact to form a quasihelical complex (25, 26), capped by the K subunit because no additional subunits could fit above it without significant steric hindrance (25) and the lack of Glu_5_ in K would not allow neutralization of the positively charged N terminus of a pilin above them. Importantly, this localization was recently confirmed by cryo-ET (27). Similarly, we found that PilA,B,C are likely to form a complex at the tip of *S. sanguinis* T4P. Although PilA is added first, because it lacks a Glu_5_, the bulky extra modules in PilB and PilC cap the PilABC complex like open wings. Due to this peculiar architecture—resembling the Winged Victory of Samothrace, a masterpiece of Greek sculpture—PilABC could only be accommodated at the tip of the T4P. Critically, both HIJK (21–23) and PilABC (34) pilins are essential for filament assembly, which is compatible with PilA being a nucleator initiating filament assembly, a role attributed to the I subunit in HIJK (28, 29). For the HIJK complex, it was proposed that i) I interacts with J in the membrane to form a staggered complex, ii) the IJ complex interacts with K via I, initiating filament assembly by forming a quasihelical complex (25), iii) the IJK complex interacts with H via J (26), and iv) the HIJK complex interacts with the major pilin via H (26). The series of events is similar for PilABC, albeit simpler. i) PilA interacts with PilC and stabilizes it [the I subunit in HIJK might play a similar role according to crystallization studies (47)], ii) PilC interacts with PilB, and iii) the PilABC complex most likely interacts with the major pilins via PilB subunit. In both systems, the I/PilA subunit initiates filament assembly and facilitates the presentation of "effectors" at the tip of filaments. In *S. sanguinis* T4P, the effectors are the two modular pilins PilB and PilC with adhesive properties. In T4P in diderm model species, the effector is the nonpilin adhesin PilC/PilY1, an extremely large protein that is absent in monoderms. Interestingly, cryo-ET analysis of *M. xanthus* T4P revealed that PilC/PilY1 forms a kinked tip structure (27), which looks like one of the open wings in PilABC.

Drawing a picture of the roles of all the pilin subunits in a model T4F system is the second major achievement in this study. *S. sanguinis* T4P are composed of five pilins: two major (PilE1, PilE2) and three minor (PilA, PilB, PilC) ones. PilE1 and PilE2—85% identical in sequence (34) —follow universally shared principles for processing by the PPase and display lollipop structures (35). This suggests that *S. sanguinis* T4P are canonical T4F, i.e., helical polymers where pilins pack within the core of the filament and are held together by interactions between their α1-helices (11). Although unusual—T4F are most often homopolymers of only one major pilin—the heteropolymeric composition of *S. sanguinis* T4P with two major pilins in nearly equal amounts (35) is not unique. Recently, the structure of a heteropolymeric archaeal T4F used for swimming by *Methanocaldococcus villosus* has been elucidated by cryo-EM (16), showing an architecture with two alternating major pilin subunits. Whether a similar alternating arrangement of PilE1 and PilE2 exists within the *S. sanguinis* T4P remains to be tested. As for the three minor pilins in *S. sanguinis* T4P they are divided into nonmodular (PilA) and modular (PilB, PilC). We have now defined their respective roles. PilA, which displays a canonical lollipop structure, interacts strongly and exclusively with PilC. Pull-down experiments suggest that PilA and PilC interact via their pilin moieties, which was confirmed by NMR. The interaction interfaces determined by NMR and predicted by modeling are in strikingly good agreement. As a functional consequence, PilA dramatically stabilizes the PilC pilin moiety, which is highly unstable on its own. Modeling suggests that this is done by β-strand complementation. PilA thus acts as an intrapilus chaperone, protecting PilC from proteolysis by perhaps participating in its folding. We previously showed that PilB displays a modular architecture with a bulky functional module grafted onto a pilin moiety (6). The vWA module in PilB makes it a bona fide adhesin, playing a key role in *S. sanguinis* adhesion to host cells by binding protein ligands such as fibronectin and fibrinogen (6). Since *S. sanguinis* T4P are critical for disease progression (48), these findings are consistent with PilB-mediated adhesion being involved in IE. Here, we show that PilC is also a modular pilin with two extra modules, an Ig-like fold followed by a ConA-like lectin/glucanase module (SSF49899). While the function of the Ig-like module remains unclear, we showed that the ConA-like module is a bona fide lectin. PilC binds specifically to two major types of glycans— i) sialylated glycans often terminated with α2-3-sialyl linkage (3′-SL or 3′-SNL), and ii) heparin oligosaccharide probes representative of sulfated GAGs—both prevalent in the human glycome (45). The measured affinities for 3′-SL and 3′-SLN are consistent with those previously determined for other SSF49899-containing proteins (49). Affinities could be much higher for larger glycan determinants, or when there is multivalent presentation (abundance) of a glycan ligand at the cell surface (45, 49). The physiological ligands for PilC are yet to be defined—identifying ligands for lectins is often difficult (45, 50)—but it is likely that this modular pilin is involved in *S. sanguinis* commensalism or IE. Indeed, 3′-SL or 3′-SLN terminated glycans are abundant in the oral cavity (32), especially on heavily glycosylated salivary mucins (45). The full picture of *S. sanguinis* T4P that has now emerged is the following. *S. sanguinis* T4P are heteropolymeric filaments composed of PilE1 and PilE2, with helical symmetry parameters that are yet to be determined, capped by a PilABC complex mediating bacterial adhesion to different eukaryotic receptors. The adhesive modules in PilB and PilC are ideally placed to maximize adhesion. However, although this is less likely, we cannot exclude the possibility that *S. sanguinis* expresses two distinct pili, capped either by PilB or PilAC.

In conclusion, by providing perhaps the most detailed structure/function characterization of the role of all pilins in a model T4P, this study sheds light on important aspects of T4F biology. The finding that PilABC might be a rudimentary version of the widely conserved HIJK strengthens the notion that complexes of minor pilins capping T4F facilitate the presentation of different effectors at the tip of filaments, thus directly promoting T4F exceptional functional versatility (1). Our findings have general implications for T4F and pave the way for further investigations that will improve our understanding of these fascinating filaments.

## Material and Methods

Detailed Material and Methods are described in the *SI Appendix*.

### Strains and Plasmids.

The *E. coli* strains and plasmids used in this study are listed in *SI Appendix*, Table S2. DH5α was used for cloning, BL21(DE3) was used for routine protein expression and purification, and B834(DE3) was used for purification of SeMet-labeled proteins.

DNA manipulations were performed using standard molecular biology techniques (51). The primers used in this study are listed in *SI Appendix*, Table S3. We amplified *pilA* directly from the *S. sanguinis* 2908 genome, while *pilB* and *pilC* were synthetic genes codon-optimized for expression in *E. coli* (GeneArt).

### Protein Purification.

The recombinant proteins were purified in two steps: affinity chromatography and SEC. 6His-tagged proteins were affinity-purified using Gravity Flow Chromatography columns (Bio-Rad) loaded with Ni-NTA agarose resin (Qiagen). Strep-tagged proteins were affinity-purified on an ÄKTA Purifier using StrepTrap HP columns (GE Healthcare). Both the 6His- and Strep-tagged proteins were then further purified by SEC on an ÄKTA Purifier using a Superdex 200 16/600 GL column (GE Healthcare), and simultaneously buffer-exchanged into 50 mM HEPES pH 7.5, 150 mM NaCl.

SeMet-labeled PilA and PilC for phasing were purified as above, but the bacteria were grown in chemically defined medium containing SeMet. ^15^N and/or ^13^C-labeled PilA for NMR analysis was purified as above, but the bacteria were grown in M9 medium supplemented with ^15^NH_4_Cl and/or ^13^C-labeled glucose.

### Protein Crystallization and Structure Determination.

Protein crystals for SeMet-labeled PilA were produced using 0.4 µL sitting drops with a 1:1 ratio protein to mother liquor (0.1 M Tris pH 8, 23% PEG 2K (w/v), 0.3 M Mg(NO_3_)_2_6H_2_O). Protein crystals for SeMet-labeled PilC were produced using 0.2 µL sitting drops with of 1:1 ratio protein to mother liquor (0.1 M Pipes pH 7, 15% w/v PEG broad smear, 0.2 M (NH_4_)_2_SO_4_, 0.01 M cadmium chloride hemi [pentahydrate)]. Data were collected at the Diamond beamlines i04-1 and i03, respectively. Data were processed using Xia2 (52), and phased using autoSHARP (53). Initial structure was produced using CRANK2 (54) and autobuild, with some manual building in Coot (55). Refinement was performed using Coot and phenix.refine (56). Structure validation was performed using MolProbity (57). Protein crystals for PilC^SK36^ were produced in 0.2 µL sitting drop with a 1:1 protein to mother liquor (0.2 M MgCl_2_6H_2_O, 20% w/v PEG 3350). Data were collected at the Diamond beamline i04, and structure was produced using the PilC structure from strain 2908 for molecular replacement.

### Pull-Down Assays.

Pull-down assays were carried out using Dynabeads His-tag Isolation and Pulldown (Invitrogen). The 6His-tagged soluble pilin domains were used as bait, while Strep-tagged soluble pilins were used as prey. The pull-down assays were performed three times for each combination. For each pull-down reaction, input, flow-through, and elution samples were analyzed by immunoblotting.

### SDS–PAGE and Immunoblotting.

SDS–PAGE was carried out using standard molecular biology techniques (51). Gels were either stained with Bio-Safe Coomassie (Bio-Rad) or transferred to a membrane and analyzed by immunoblotting. We used specific antibodies generated in rabbits against PilE1, PilE2, PilA, PilB, and PilC, which were previously described (34, 35). The secondary was an anti-rabbit antibody conjugated to horseradish peroxidase (GE Healthcare).

### SEC–MALS.

SEC–MALS was performed using an ÄKTA Prime system with a S200 10/300 GL column (GE Healthcare) and a MALS detector (Wyatt). The UV, light scattering and refractive index signals were analyzed with the ASTRA chromatography software.

### ITC.

ITC was used to study the interaction between the minor pilins PilA and PilC, as well as the binding of PilC_Δpilin_ to sugar ligands. The experiments were performed on a MicroCal ITC200 Malvern machine. Each experiment was repeated three times. Data analysis was performed on the Origin ITC200 software. Statistical analyses were performed with Prism (GraphPad Software). Comparisons were done by one-way ANOVA, followed by Dunnett’s multiple comparison tests with 99% CI. An adjusted *P* value < 0.01 was considered statistically significant (**P < 0.01, ***P < 0.001, ****P < 0.0001).

### NMR Assignment and Chemical Shift Perturbations.

We performed TROSY-based assignment experiments: HNCA, HNCOCA, HNCO, HNCACO, HNCACB, and CBCACONH. All data were collected at 25 °C on a Bruker Avance III HD 800MHz triple resonance spectrometer with a cryoprobe.

Chemical shift perturbation experiments were performed with samples in NMR buffer containing ^15^N-labeled PilA with or without unlabeled PilC. Chemical shift perturbations were determined using the equation:

$$\Delta \delta =\sqrt{{\left(\Delta \delta_{\text{H}}\right)}^{2}+\;{\left(\frac{{\text{γ}}_{\text{N}}}{{\text{γ}}_{\text{H}}}\Delta {\text{γ}}_{\text{N}}\right)}^{2}}.$$

### Protein Stability Assays.

To perform protein stability tests, PilA and PilC were purified and mixed at equal concentrations. In long-term assays, the proteins were kept at 4 °C for 4 wk. Samples were taken once a week and analyzed by SDS–PAGE/Coomassie staining. In trypsin sensitivity assays, the proteins were incubated with trypsin (Sigma) for 60 min on ice. Samples—taken at 1, 5, 10, 20, 40, and 60 min—were analyzed by SDS–PAGE/Coomassie staining.

### Glycan Microarrays.

The binding specificities of 6His–PilC, 6His–PilC_Δpilin_ and 6His–PilC^SK36^ were analyzed using a NGL-based microarray system (42). A previously described broad-spectrum microarray of 672 sequence-defined lipid-linked glycan probes was used (58). The list of glycan probes is given in the Dataset S1. Details of the preparation of the glycan probes and the generation of the microarrays are listed in *SI Appendix*, Table S4 in accordance with the MIRAGE guidelines (59). The microarray analyses were performed essentially as described (60). Binding was detected with Alexa Fluor-647-labeled streptavidin (Molecular Probes). Imaging and data analysis are described in *SI Appendix*, Table S4.

### Bioinformatics and Modeling.

This was done as previously described (6). Modeling was done using AlphaFold 2.2.0 (38) and AlphaFold-Multimer (61). Five predictions were generated in total, with a final relaxation step. The predictions were ranked according to IPTM+PTM and the top predictions were chosen.

## Supplementary Material

Appendix 01 (PDF)

Dataset S01 (XLSX)

## Data Availability

Protein 3D structures data have been deposited in PDB (7O5Y, 7OA7, 7OA8) (62–64). The PilABC structural model is available in ModelArchive (https://modelarchive.org) with the accession code ma-zz3r2 (65). All study data are included in the article and/or *SI Appendix*.

## References

[1] J. L. Berry, V. Pelicic, Exceptionally widespread nano-machines composed of type IV pilins: the prokaryotic Swiss Army knives. FEMS Microbiol. Rev. **39**, 134–154 (2015). 25793961 10.1093/femsre/fuu001 PMC4471445

[2] R. Denise, S. S. Abby, E. P. C. Rocha, Diversification of the type IV filament superfamily into machines for adhesion, protein secretion, DNA uptake, and motility. PLoS Biol. **17**, e3000390 (2019). 31323028 10.1371/journal.pbio.3000390 PMC6668835

[3] Y. W. Chang , Architecture of the type IVa pilus machine. Science **351**, aad2001 (2016). 26965631 10.1126/science.aad2001 PMC5929464

[4] C. L. Giltner, Y. Nguyen, L. L. Burrows, Type IV pilin proteins: Versatile molecular modules. Microbiol. Mol. Biol. Rev. **76**, 740–772 (2012). 23204365 10.1128/MMBR.00035-12 PMC3510520

[5] Z. Szabó , Identification of diverse archaeal proteins with class III signal peptides cleaved by distinct archaeal prepilin peptidases. J. Bacteriol. **189**, 772–778 (2007). 17114255 10.1128/JB.01547-06 PMC1797317

[6] C. Raynaud, D. Sheppard, J. L. Berry, I. Gurung, V. Pelicic, PilB from *Streptococcus sanguinis* is a bimodular type IV pilin with a direct role in adhesion. Proc. Natl. Acad. Sci. U.S.A. **118**, e2102092118 (2021). 34031252 10.1073/pnas.2102092118 PMC8179133

[7] M. R. Kaufman, J. M. Seyer, R. K. Taylor, Processing of TCP pilin by TcpJ typifies a common step intrinsic to a newly recognized pathway of extracellular protein secretion by Gram-negative bacteria. Genes Dev. **5**, 1834–1846 (1991). 1680773 10.1101/gad.5.10.1834

[8] D. N. Nunn, S. Lory, Product of the *Pseudomonas aeruginosa* gene *pilD* is a prepilin leader peptidase. Proc. Natl. Acad. Sci. U.S.A. **88**, 3281–3285 (1991). 1901657 10.1073/pnas.88.8.3281 PMC51430

[9] C. F. LaPointe, R. K. Taylor, The type 4 prepilin peptidases comprise a novel family of aspartic acid proteases. J. Biol. Chem. **275**, 1502–1510 (2000). 10625704 10.1074/jbc.275.2.1502

[10] M. McCallum, S. Tammam, A. Khan, L. L. Burrows, P. L. Howell, The molecular mechanism of the type IVa pilus motors. Nat. Commun. **8**, 15091 (2017). 28474682 10.1038/ncomms15091 PMC5424180

[11] E. H. Egelman, Cryo-EM of bacterial pili and archaeal flagellar filaments. Curr. Opin. Struct. Biol. **46**, 31–37 (2017). 28609682 10.1016/j.sbi.2017.05.012 PMC5683938

[12] S. Kolappan , Structure of the *Neisseria meningitidis* type IV pilus. Nat. Commun. **7**, 13015 (2016). 27698424 10.1038/ncomms13015 PMC5059446

[13] F. Wang , Cryoelectron microscopy reconstructions of the *Pseudomonas aeruginosa* and *Neisseria gonorrhoeae* type IV pili at sub-nanometer resolution. Structure **25**, 1423–1435 (2017). 28877506 10.1016/j.str.2017.07.016 PMC8189185

[14] A. Lopez-Castilla , Structure of the calcium-dependent type 2 secretion pseudopilus. Nat. Microbiol. **2**, 1686–1695 (2017). 28993624 10.1038/s41564-017-0041-2 PMC5705324

[15] A. Luna Rico, W. Zheng, N. Petiot, E. H. Egelman, O. Francetic, Functional reconstitution of the type IVa pilus assembly system from enterohaemorrhagic *Escherichia coli*. Mol. Microbiol. **111**, 732–749 (2019). 30561149 10.1111/mmi.14188 PMC6417937

[16] L. Gambelli , An archaellum filament composed of two alternating subunits. Nat. Commun. **13**, 710 (2022). 35132062 10.1038/s41467-022-28337-1 PMC8821640

[17] A. Cehovin , Specific DNA recognition mediated by a type IV pilin. Proc. Natl. Acad. Sci. U.S.A. **110**, 3065–3070 (2013). 23386723 10.1073/pnas.1218832110 PMC3581936

[18] J. L. Berry , A comparative structure/function analysis of two type IV pilin DNA receptors defines a novel mode of DNA binding. Structure **24**, 926–934 (2016). 27161979 10.1016/j.str.2016.04.001 PMC4906244

[19] J. P. Barnier , The minor pilin PilV provides a conserved adhesion site throughout the antigenically variable meningococcal type IV pilus. Proc. Natl. Acad. Sci. U.S.A. **118** (2021). 10.1073/pnas.2109364118 PMC8609321 34725157

[20] S. Helaine, D. H. Dyer, X. Nassif, V. Pelicic, K. T. Forest, 3D structure/function analysis of PilX reveals how minor pilins can modulate the virulence properties of type IV pili. Proc. Natl. Acad. Sci. U.S.A. **104**, 15888–15893 (2007). 17893339 10.1073/pnas.0707581104 PMC2000383

[21] H. C. Winther-Larsen , *Neisseria gonorrhoeae* PilV, a type IV pilus-associated protein essential to human epithelial cell adherence. Proc. Natl. Acad. Sci. U.S.A. **98**, 15276–15281 (2001). 11752467 10.1073/pnas.261574998 PMC65020

[22] E. Carbonnelle, S. Helaine, L. Prouvensier, X. Nassif, V. Pelicic, Type IV pilus biogenesis in *Neisseria meningitidis*: PilW is involved in a step occuring after pilus assembly, essential for fiber stability and function. Mol. Microbiol. **55**, 54–64 (2005). 15612916 10.1111/j.1365-2958.2004.04364.x

[23] C. L. Giltner, M. Habash, L. L. Burrows, *Pseudomonas aeruginosa* minor pilins are incorporated into type IV pili. J. Mol. Biol. **398**, 444–461 (2010). 20338182 10.1016/j.jmb.2010.03.028

[24] D. A. Cisneros, G. Pehau-Arnaudet, O. Francetic, Heterologous assembly of type IV pili by a type II secretion system reveals the role of minor pilins in assembly initiation. Mol. Microbiol. **86**, 805–818 (2012). 23006128 10.1111/mmi.12033

[25] K. V. Korotkov, W. G. Hol, Structure of the GspK-GspI-GspJ complex from the enterotoxigenic *Escherichia coli* type 2 secretion system. Nat. Struct. Mol. Biol. **15**, 462–468 (2008). 18438417 10.1038/nsmb.1426

[26] C. A. Escobar , Structural interactions define assembly adapter function of a type II secretion system pseudopilin. Structure **29**, 1116–1127 (2021). 34139172 10.1016/j.str.2021.05.015

[27] A. Treuner-Lange , PilY1 and minor pilins form a complex priming the type IVa pilus in *Myxococcus xanthus*. Nat. Commun. **11**, 5054 (2020). 33028835 10.1038/s41467-020-18803-z PMC7541494

[28] D. A. Cisneros, P. J. Bond, A. P. Pugsley, M. Campos, O. Francetic, Minor pseudopilin self-assembly primes type II secretion pseudopilus elongation. EMBO J. **31**, 1041–1053 (2012). 22157749 10.1038/emboj.2011.454 PMC3280553

[29] B. Douzi , The XcpV/GspI pseudopilin has a central role in the assembly of a quaternary complex within the T2SS pseudopilus. J. Biol. Chem. **284**, 34580–34589 (2009). 19828448 10.1074/jbc.M109.042366 PMC2787320

[30] S. Melville, L. Craig, Type IV pili in Gram-positive bacteria. Microbiol. Mol. Biol. Rev. **77**, 323–341 (2013). 24006467 10.1128/MMBR.00063-12 PMC3811610

[31] V. Pelicic, Monoderm bacteria: The new frontier for type IV pilus biology. Mol. Microbiol. **112**, 1674–1683 (2019). 31556183 10.1111/mmi.14397 PMC6916266

[32] J. Kreth, R. A. Giacaman, R. Raghavan, J. Merritt, The road less traveled–Defining molecular commensalism with *Streptococcus sanguinis*. Mol. Oral. Microbiol. **32**, 181–196 (2017). 27476770 10.1111/omi.12170 PMC5288394

[33] T. J. Cahill, B. D. Prendergast, Infective endocarditis. Lancet **387**, 882–893 (2016). 26341945 10.1016/S0140-6736(15)00067-7

[34] I. Gurung , Functional analysis of an unusual type IV pilus in the Gram-positive *Streptococcus sanguinis*. Mol. Microbiol. **99**, 380–392 (2016). 26435398 10.1111/mmi.13237 PMC4832360

[35] J. L. Berry , Global biochemical and structural analysis of the type IV pilus from the Gram-positive bacterium *Streptococcus sanguinis*. J. Biol. Chem. **294**, 6796–6808 (2019). 30837269 10.1074/jbc.RA118.006917 PMC6497953

[36] S. Imam, Z. Chen, D. S. Roos, M. Pohlschröder, Identification of surprisingly diverse type IV pili, across a broad range of Gram-positive bacteria. PLoS One **6**, e28919 (2011). 22216142 10.1371/journal.pone.0028919 PMC3244431

[37] L. Craig , Type IV pilin structure and assembly. X-ray and EM analyses of *Vibrio cholerae* toxin-coregulated pilus and *Pseudomonas aeruginosa* PAK pilin. Mol. Cell **11**, 1139–1150 (2003). 12769840 10.1016/s1097-2765(03)00170-9

[38] J. Jumper , Highly accurate protein structure prediction with AlphaFold. Nature **596**, 583–589 (2021). 34265844 10.1038/s41586-021-03819-2 PMC8371605

[39] P. Bork, L. Holm, C. Sander, The immunoglobulin fold. Structural classification, sequence patterns and common core. J. Mol. Biol. **242**, 309–320 (1994). 7932691 10.1006/jmbi.1994.1582

[40] E. Wong , The Vibrio cholerae colonization factor GbpA possesses a modular structure that governs binding to different host surfaces. PLoS Pathog. **8**, e1002373 (2012). 22253590 10.1371/journal.ppat.1002373 PMC3257281

[41] D. D. Leonidas , Structural basis for the recognition of carbohydrates by human galectin-7. Biochemistry **37**, 13930–13940 (1998). 9760227 10.1021/bi981056x

[42] Y. Liu , Neoglycolipid-based oligosaccharide microarray system: preparation of NGLs and their noncovalent immobilization on nitrocellulose-coated glass slides for microarray analyses. Meth. Mol. Biol. **808**, 117–136 (2012). 10.1007/978-1-61779-373-8_8 22057521

[43] W. L. Marsh , Anti-Sdx: A "new" auto-agglutinin related to the Sda blood group. Transfusion **20**, 1–8 (1980). 7355457 10.1046/j.1537-2995.1980.20180125021.x

[44] J. A. Morton, M. M. Pickles, A. M. Terry, The Sda blood group antigen in tissues and body fluids. Vox Sang. **19**, 472–482 (1970). 5503169 10.1111/j.1423-0410.1970.tb01779.x

[45] R. D. Cummings, The repertoire of glycan determinants in the human glycome. Mol. Biosyst. **5**, 1087–1104 (2009). 19756298 10.1039/b907931a

[46] V. Pelicic, Type IV pili: *e pluribus unum*? Mol. Microbiol. **68**, 827–837 (2008). 18399938 10.1111/j.1365-2958.2008.06197.x

[47] M. E. Yanez, K. V. Korotkov, J. Abendroth, W. G. Hol, The crystal structure of a binary complex of two pseudopilins: EpsI and EpsJ from the type 2 secretion system of *Vibrio vulnificus*. J. Mol. Biol. **375**, 471–486 (2008). 18022192 10.1016/j.jmb.2007.10.035 PMC2219201

[48] A. M. Martini, B. S. Moricz, L. J. Woods, B. D. Jones, Type IV pili of *Streptococcus sanguinis* contribute to pathogenesis in experimental infective endocarditis. Microbiol. Spectr. **9**, e0175221 (2021). 34756087 10.1128/Spectrum.01752-21 PMC8579931

[49] S. Böcker, L. Elling, Binding characteristics of galectin-3 fusion proteins. Glycobiology **27**, 457–468 (2017). 28104787 10.1093/glycob/cwx007

[50] P. R. Crocker, T. Feizi, Carbohydrate recognition systems: Functional triads in cell-cell interactions. Curr. Opin. Struct. Biol. **6**, 679–691 (1996). 8913692 10.1016/s0959-440x(96)80036-4

[51] J. Sambrook, D. W. Russell, Molecular Cloning. A Laboratory Manual (Cold Spring Harbor Laboratory Press, Cold Spring Harbor, New York, 2001).

[52] G. Winter, C. M. Lobley, S. M. Prince, Decision making in *xia*2. Acta Crystallogr. Sect. D. Biol. Crystallogr. **69**, 1260–1273 (2013). 23793152 10.1107/S0907444913015308 PMC3689529

[53] C. Vonrhein, E. Blanc, P. Roversi, G. Bricogne, Automated structure solution with autoSHARP. Meth. Mol. Biol. **364**, 215–230 (2007). 10.1385/1-59745-266-1:215 17172768

[54] P. Skubak , A new MR-SAD algorithm for the automatic building of protein models from low-resolution X-ray data and a poor starting model. IUCrJ **5**, 166–171 (2018). 10.1107/S2052252517017961 PMC5947721 29765606

[55] P. Emsley, B. Lohkamp, W. G. Scott, K. Cowtan, Features and development of Coot. Acta Crystallogr. Sect. D. Biol. Crystallogr. **66**, 486–501 (2010). 20383002 10.1107/S0907444910007493 PMC2852313

[56] P. V. Afonine , Towards automated crystallographic structure refinement with phenix.refine. Acta Crystallogr. Sect. D. Biol. Crystallogr. **68**, 352–367 (2012). 22505256 10.1107/S0907444912001308 PMC3322595

[57] V. B. Chen , MolProbity: All-atom structure validation for macromolecular crystallography. Acta Crystallogr. Sect. D. Biol. Crystallogr. **66**, 12–21 (2010). 20057044 10.1107/S0907444909042073 PMC2803126

[58] N. McAllister , Chikungunya virus strains from each genetic clade bind sulfated glycosaminoglycans as attachment factors. J. Virol. **94**, e01500–01520 (2020). 32999033 10.1128/JVI.01500-20 PMC7925169

[59] Y. Liu , The minimum information required for a glycomics experiment (MIRAGE) project: Improving the standards for reporting glycan microarray-based data. Glycobiology **27**, 280–284 (2017). 27993942 10.1093/glycob/cww118 PMC5444268

[60] U. Neu , A structure-guided mutation in the major capsid protein retargets BK polyomavirus. PLoS Pathog. **9**, e1003688 (2013). 24130487 10.1371/journal.ppat.1003688 PMC3795024

[61] R. Evans , Protein complex prediction with AlphaFold-multimer. bioRxiv [Preprint] (2022). https://www.biorxiv.org/content/10.1101/2021.10.04.463034v2 (Accessed 22 December 2022).

[62] D. Sheppard, V. Pelicic, 7O5Y, Protein Data Bank. https://www.rcsb.org/structure/7O5Y. Deposited 9 April 2021.

[63] D. Sheppard, V. Pelicic, 7OA7, Protein Data Bank.https://www.rcsb.org/structure/7OA7. Deposited 19 April 2021.

[64] D. Sheppard, V. Pelicic, 7OA8, Protein Data Bank.https://www.rcsb.org/structure/7OA8. Deposited 19 April 2021.

[65] V. Pelicic, ma-zz3r2, ModelArchive. https://www.modelarchive.org/doi/10.5452/ma-zz3r2. Accessed 22 December 2022.

