# Supplementary material for: Characterization of a glycan-binding complex of minor pilins completes the analysis of Streptococcus sanguinis type 4 pili subunits
Source: Proc Natl Acad Sci U S A. 2023 Jan 10;120(3):e2216237120. doi: 10.1073/pnas.2216237120 (PMC9934059; doi:10.1073/pnas.2216237120)
Supplement: Supplementary file 1 — Appendix 01 (PDF) [file pnas.2216237120.sapp.pdf]

Supporting Information for

Characterisation of a glycan-binding complex of minor pilins completes the analysis of *Streptococcus sanguinis* type 4 pili subunits

Meriam Shahin[a,1], Devon Sheppard[a,1], Claire Raynaud[a], Jamie-Lee Berry[a], Ishwori Gurung[a], Lisete M. Silva[b], Ten Feizi[b], Yan Liu[b], Vladimir Pelicic[a,c,*]

[a]MRC Centre for Molecular Bacteriology and Infection, Imperial College London, London, United Kingdom

[b]Glycosciences Laboratory, Department of Metabolism, Digestion and Reproduction, Imperial College London, London, United Kingdom

[c]Laboratoire de Chimie Bactérienne, UMR 7283 CNRS/Aix-Marseille Université, Institut de Microbiologie de la Méditerranée, Marseille, France

[1]These authors contributed equally

*Corresponding author
email: vladimir.pelicic@inserm.fr

This PDF file includes:

Supporting text
Figures S1 to S8
Tables S1 to S4
Legends for Dataset S1
SI References

Other supporting materials for this manuscript include the following:

Dataset S1

Materials and methods

Strains and growth conditions

E. coli strains were grown in liquid or solid lysogeny broth (LB) medium (Difco), containing 50 µg/ml kanamycin (Sigma) when required. *E. coli* B834(DE3) was grown in chemically defined medium (CDM) supplemented with 20 mg/ml SeMet (Sigma).

Chemically competent cells were prepared as described elsewhere (1). All PCR were done using high-fidelity DNA polymerases (Agilent). Missense mutations in recombinant proteins were generated by QuikChange site-directed mutagenesis (Agilent) using the respective pET28b derivatives as templates. To purify PilA and PilC, the soluble portion of these proteins (excluding the leader peptide and the predicted α1N helix) was fused to non-cleavable N-terminal 6His or Strep II tags and cloned into pET-28b (Novagen).

Protein purification

The respective pET-28b derivatives were transformed into *E. coli* BL21(DE3). A single colony was picked and grown O/N in LB with kanamycin. The following morning, this culture was back diluted 1/100 in 1 l of the same medium. Once the OD_{600} (optical density at 600 nm) reached 0.8-1, the cultures were cooled down to 16°C, and protein expression was induced O/N with 0.5 mM isopropyl 1-thio ß-D-galactopyranoside (IPTG) (Merck Chemicals). The next day, the cells were harvested by centrifugation at 6,000 *g* for 20 min, and the pellets were frozen at -80°C in the appropriate binding buffer, containing the SigmaFast EDTA-free protease inhibitor cocktail (Sigma). Cells were lysed by repeated cycles of sonication (5 sec on/off pulses for 3-5 min) and the clarified cell lysate was prepared by centrifugation at 11,000 *g* for 30 min.

6His-tagged proteins were affinity-purified using Gravity Flow Chromatography columns (Bio-Rad) loaded with 1-2 ml Ni-NTA agarose resin (Qiagen) pre-

equilibrated with binding buffer. The resin was mixed with the cell lysate and the column was allowed to drain. The column was then washed with binding buffer (50 mM HEPES pH 7.5, 150 mM NaCl, 20 mM imidazole) several times, before being eluted with elution buffer (50 mM HEPES pH 7.5, 150 mM NaCl, 300 mM imidazole). Strep-tagged proteins were affinity-purified on an ÄKTA Purifier using 5 ml StrepTrap HP columns (GE Healthcare) according to manufacturer's instructions. The columns were washed with binding buffer (50 mM HEPES pH 7.5, 150 mM NaCl) and protein was eluted with elution buffer (50 mM HEPES pH 7.5, 150 mM NaCl, 2.5 mM desthiobiotin). Both the 6His- and Strep-tagged proteins were then further purified by SEC on an ÄKTA Purifier using a Superdex 200 16/600 GL column (GE Healthcare), and simultaneously buffer-exchanged into 50 mM HEPES pH 7.5, 150 mM NaCl. Protein concentration was quantified spectrophotometrically on a NanoDrop Lite (Thermo Fisher Scientific).

To purify SeMet-labelled PilA and PilC for phasing, the corresponding pET-28b derivatives were transformed in *E. coli* B834(DE3). A single colony was picked and grown O/N in LB with kanamycin. After back dilution, transformants were grown at 37°C in selective liquid LB, until OD_{600} reached 0.6-0.7. The cells were pelleted at 8,000 *g* for 5 min and washed twice with 2 ml of CDM containing no Met. The pellets were then washed with 2 ml of CDM supplemented with 20 mg/ml L-Met (Sigma) and used to inoculate, at 1/200 dilution, 20 ml of CDM supplemented with 20 mg/ml Met. These cultures were grown O/N at 37°C. Cells were pelleted and washed three times with CDM. Then, the pellets were re-suspended in 20 ml of CDM, supplemented with 20 mg/ml SeMet, and used to inoculate 1 l of CDM supplemented with SeMet. Cells were grown at 37°C until OD_{600} reached 0.5-0.7. The cultures were cooled down to 16°C, and protein expression was induced by adding 1 mM IPTG and 4 ml of 36 % glucose (w/v). The cultures were further supplemented with another 4 ml of 36 % glucose 2.5 h later. The next day, the cells were harvested, and SeMet-labelled proteins were purified as above.

To purify ^{15}N and/or ^{13}C-labelled PilA for NMR analysis, the corresponding pET-28b derivative was transformed in *E. coli* BL21(DE3). A single colony was used to inoculate a starter culture, which was back-diluted 1/100 into 1 l kanamycin-supplemented LB the following morning. The cultures were grown at 37°C until the OD_{600} reached 0.9. The cells were harvested by centrifugation at 6,000 *g* for 10 min at 4°C. The pelleted cells were resuspended in 490 ml M9 salts (3.37 mM Na_2HPO_4. 2.2 mM KH_2PO_4, 0.855 mM NaCl, pH 7.2), containing 1 ml 1 M $MgSO_4$ and 250 µl 0.2 M $CaCl_2$. The cell suspension was supplemented with 5 mg vitamin B1, 50 µg/ml kanamycin, 0.5 g $^{15}NH_4Cl$ and 2 g unlabelled D-glucose (or ^{13}C-labelled glucose if double isotopic labelling was required) dissolved in 10 ml H_2O and filter-sterilised. The cells were rested at 16°C for 20 min before protein expression was induced O/N with 0.5 mM IPTG. The cells were harvested, lysed, and purified as described above. SEC was performed in NMR buffer (10 mM Na_2HPO_4/NaH_2PO_4 pH 7.0, 150 mM NaCl).

Pull-down assays

Pull-down assays were carried out using Dynabeads His-tag Isolation and Pulldown (Invitrogen). The 6His-tagged soluble pilin domains were used as bait, while Strep-tagged soluble pilins were used as prey. For each pull-down reaction, 25 µl of magnetic beads and 1.5 nmol of protein were used (23 µg for PilA, 75 µg for PilC, and 60 µg $PilC_{\Delta pilin}$). The pull-down assays were performed three times for each combination. Prior to the pull-down assays, the bait and prey proteins were mixed in 1 ml of binding buffer (50 mM HEPES pH 7.6, 150 mM NaCl, 10 mM imidazole, 0.1 % Tween-20) and incubated on ice for 1 h. Meanwhile, the magnetic beads were incubated with 700 µl blocking buffer (50 mM HEPES pH 7.6, 150 mM NaCl, 10 mM imidazole, 0.1 % Tween-20, 5 % skim milk) on a rotating wheel at 4°C. After 1 h, the blocking reaction was placed on a magnet for 10 sec to capture the beads, and the flow-through was discarded. The beads were then rinsed twice with 700 µl binding

buffer and mixed with 1 ml of bait-prey reaction mixture. Following a 20-min incubation on a rotating wheel at 4°C, the beads were captured on the side of the tube using a magnet, and the flow-through was removed. Beads were washed 10 times by vortexing them with 500 µl washing buffer (50 mM HEPES pH 7.6, 150 mM NaCl, 20 mM imidazole, 0.1 % Tween-20) for 10 sec. After the final wash, the beads were incubated with 100 µl elution buffer (50 mM HEPES pH 7.6, 300 mM NaCl, 500 mM imidazole, 0.1 % Tween-20) for 5 min on a rotating wheel at 4°C. The beads were captured on the side of the tube using a magnet, and the flow-through was carefully transferred to a fresh tube. For each pull-down reaction, input, flow-through, and elution samples were analysed. The samples were mixed with 2x Laemmli Buffer (Bio-Rad), boiled for 5 min at 100°C, and subsequently analysed by immunoblotting.

SDS-PAGE and immunoblotting

SDS-PAGE was carried out using 1x Tris/Glycine/SDS Buffer (Bio-Rad) in a Mini-Protean Tetra cell system (Bio-Rad) for 1 h at 200 V. The Precision Plus Protein All Blue Prestained Protein Standards (Bio-Rad) was used as molecular weight marker and loaded alongside the protein samples. Gels were either stained with Bio-Safe Coomassie (Bio-Rad) and imaged with a Gel Doc EZ Imager (Bio-Rad) or transferred to a membrane and analysed by immunoblotting.

Immunoblotting was done as follows. After proteins were separated by SDS-PAGE, they were transferred onto Amersham Hybond ECL nitrocellulose membrane (GE Healthcare). The wet transfer was carried out for 1 h at 100 V in ice-cold buffer (39 mM glycine, 48 mM Tris base, 0.037 % SDS, 20 % isopropanol). The blotted membranes were blocked for 1 h at room temperature, while shaking, in PBS supplemented with 0.1 % Tween-20 (PBST) containing 5 % (w/v) skim milk powder (VWR). The membranes were then incubated for 1 h with primary antibodies diluted 1/3,000 in 5 % milk PBST. The specific antibodies generated in rabbits against PilE1, PilE2, PilA, PilB, PilC, were previously described (2, 3). Following three 10-min

washes with PBST, the membranes were incubated for 1 h with an anti-rabbit secondary antibody conjugated to horseradish peroxidase (GE Healthcare), diluted 1/10,000 in PBST. The membranes were washed again three times for 10 min in PBST, dried and developed with Amersham ECL Prime Western Blotting Detection Reagent (GE Healthcare). Protein bands were detected using a ChemiDoc Imaging System (Bio-Rad).

SEC-MALS

SEC-MALS was performed using an ÄKTA Prime system with a S200 10/300 GL column (GE Healthcare) and a MALS detector (Wyatt). The column and the system were equilibrated in buffer (50 mM HEPES pH 7.5, 150 mM NaCl) for 48-72 h to minimise and stabilise the light scattering and background noise. Protein samples of 150 µl (45 µM PilA, 50 µM PilC, 40 µM PilA-PilC) were loaded onto the column at 0.2 ml/min.

ITC

For measuring the affinity of the PilA-PilC interaction, the two purified proteins were buffer-exchanged into the same buffer (20 mM HEPES pH 7.5, 150 mM NaCl). The sample cell was loaded with 20 µM PilC, while the syringe was filled with 200 µM PilA. Each ITC run consisted of 20 titrations in total – the first injection was 0.4 µl, while all subsequent injections were 1.9 µl in volume. There was a 200-sec-long gap between each titration. To quantify the protein-sugar interactions, the purified $PilC_{\Delta pilin}$ and the 3’-SL and 3’-SLN sugar ligands (Dextra Laboratories) were prepared in the same buffer (20 mM HEPES pH 7.6, 50 mM NaCl). The sample cell was loaded with 0.1 mM purified protein, and the syringe was filled with 2 mM of glycan ligand. Each ITC run consisted of 30 titrations in total with a 150-sec-long gap between each titration. The first titration was 0.4 µl, while all subsequent titrations were 1.2 µl in volume.

NMR assignment and chemical shift perturbations

A sample containing ^{13}C, ^{15}N labelled PilA at 1 mM in NMR buffer (10 mM Na_2HPO_4/NaH_2PO_4 pH 7, 150 mM NaCl, 5 % D_2O) was used for the TROSY-based assignment experiments. Experiments were processed using MddNMR (4) for reconstruction after Non-Uniform Sampling, and NMRPipe (5). Peak picking and assignments were performed in SPARKY (6).

Chemical shift perturbation experiments were performed using TROSY-based HSQC experiments with samples containing either ^{15}N labelled PilA at 1 mM (in NMR buffer) or ^{15}N labelled PilA at 1 mM and unlabelled PilC at 0.5 mM (in NMR buffer).

Protein stability assays

To perform long-term protein stability tests, 6His-PilA and 6His-PilC were purified and mixed at 400 μM concentration. The single proteins and the complex – three independent aliquots for each – were kept at 4°C for four weeks. Samples were taken once a week, mixed with 2x Laemmli buffer and analysed by SDS-PAGE/Coomassie staining to reveal protein degradation.

To perform trypsin sensitivity assays, 6His-PilA and 6His-PilC were purified and one hundred μl aliquots of PilA, PilC and the PilA-PilC complex were prepared at 80 μM concentration. The PilA-PilC complex was incubated at 4°C O/N. The next day, the three aliquots were incubated with trypsin (Sigma) at 1/1,000 dilution for 60 min on ice. Samples of 10 μl were taken at 1, 5, 10, 20, 40 and 60 min. The samples were immediately mixed with 2x Laemmli buffer, boiled at 100°C and analysed by SDS-PAGE/Coomassie staining. The trypsin sensitivity assays were performed three times with freshly purified proteins.

Glycan microarrays

The binding specificities of 6His-PilC, 6His-PilC$_{\Delta pilin}$ and 6His-PilC^{SK36} – purified and concentrated to 1 mg/ml in 10 mM HEPES pH 7.5, 150 mM NaCl, 5 mM $CaCl_2$ – were analysed using a NGL-based microarray system (7). The list of glycan probes is given in the Dataset S1. Details of the preparation of the glycan probes and the generation of the microarrays are listed in Table S4. The microarray analyses were performed essentially as described (8). In brief, after blocking of the slides for 1 h with HBS buffer (10 mM HEPES pH 7.4, 150 mM NaCl) containing 1 % (w/v) BSA (Sigma), 0.02 % (w/v) Casein (Pierce), and 10 mM CaCl2, the 6His-tagged PilC proteins were analysed under two conditions. In condition A, the microarrays were overlaid with the 6His-PilC proteins for 90 min as precomplexed protein-antibody complexes. These were prepared by preincubating the His-tagged PilC with mouse monoclonal anti-poly-histidine and biotinylated anti-mouse IgG antibodies (both from Sigma) at a ratio of 1:1.5:1.5 (by weight) and diluted in the blocking solution to provide a final PilC concentration of 50 µg/ml. In condition B, the microarrays were first overlaid with the 6His-PilC proteins at 100 µg/ml. This was followed by incubation with mouse monoclonal anti-His and biotinylated anti-mouse IgG antibodies (both at 10 µg/ml). In both conditions, binding was detected with Alexa Fluor-647-labelled streptavidin (Molecular Probes) at 1 µg/ml for 30 min. All steps were carried out at ambient temperature except for the precomplexation step which was carried out on ice. Imaging and data analysis are described in Table S4.

Bioinformatics and modelling

Protein sequences were routinely analysed using DNA Strider (9). Prediction of protein domains was done by interrogating the InterPro database with InterProScan (10). Molecular visualisation of 3D structures was done using PyMOL (Schrödinger), which was used for generating the figures in this manuscript. The DALI server was used for comparing protein structures in 3D (11). Protein 3D structures were downloaded from the RCSB PDB server. The 3d-SS (12) server was used to

superpose 3D protein structures with the STAMP algorithm (13). PDBePISA (14) was used for the exploration of macromolecular interfaces. Modelling was done using AlphaFold (15) and AlphaFold-Multimer (16).

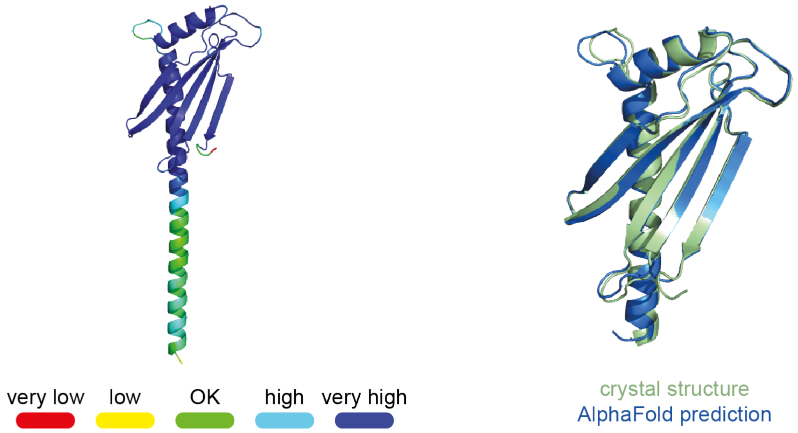

Fig. S1. AlphaFold model of PilA. Left, full-length model coloured following B-factor accuracy. Right, superposition of our crystal structure (green) with the corresponding portion of the AlphaFold (15) prediction (blue). The two structures superpose with an RMSD of 0.58 Å, showing structural identity.

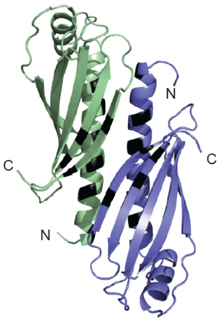

H-bond	PilA 1	dist. (Å)	PilA 2
1	THR_{31}	3.49	TRP_{52}
2	TYR_{37}	2.83	HIS_{133}
3	TYR_{37}	2.56	GLU_{147}
4	LYS_{41}	2.85	TYR_{131}
5	THR_{45}	3.55	TYR_{42}
6	GLN_{49}	2.62	TYR_{42}
7	TRP_{52}	3.38	THR_{31}
8	TYR_{131}	2.68	LYS_{41}
9	HIS_{133}	2.84	TYR37
10	GLU_{147}	2.49	TYR_{37}
11	GLU_{147}	2.88	LEU_{151}
12	LEU_{151}	2.88	GLU_{147}

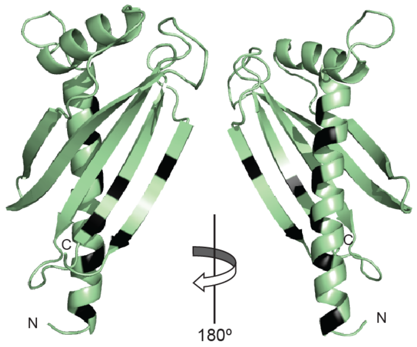

Fig. S2. Interaction interface in PilA dimers. Left, head-to-toe dimers in the crystal are stabilised by a series of hydrogen bonds between residues in α1-helix and the last 2 β-strands (black). Numbering is according to the mature pilin. Right, Close-up views at 180º rotation of one of the monomers in the dimer.

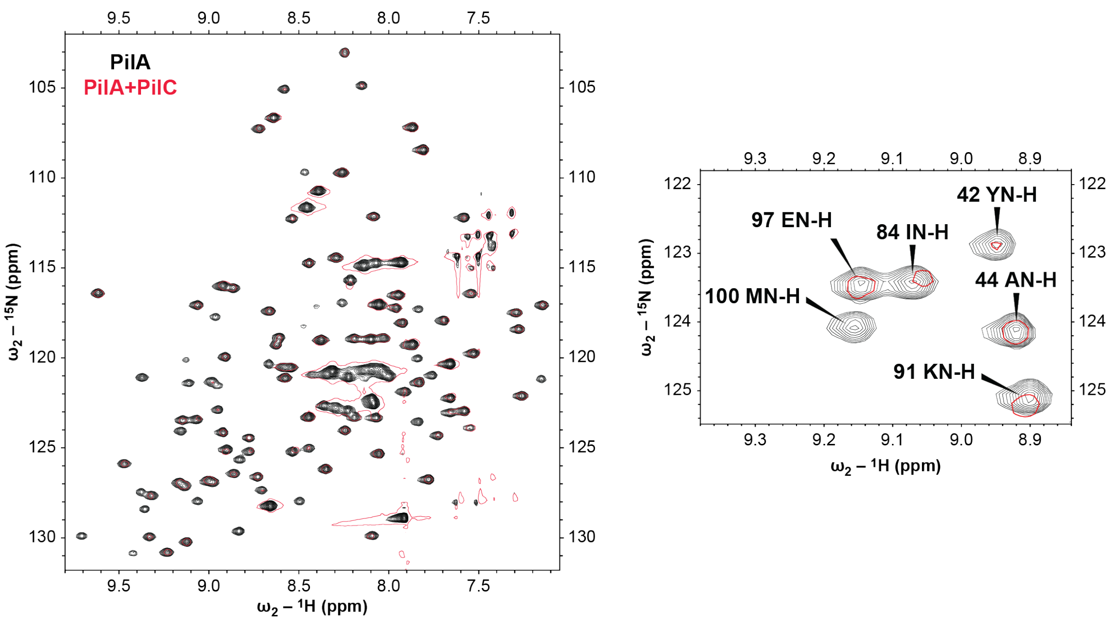

Fig. S3. NMR analysis of PilA ± PilC. Left, region of the ^{1}H-^{15}N HSQC spectrum of PilA. Free PilA spectrum is in black. A simplified PilA spectrum in the presence of PilC is in red. Right, close-up in an area of PilA showing significant amide shift changes in the presence of PilC. Numbering is according to the mature pilin.

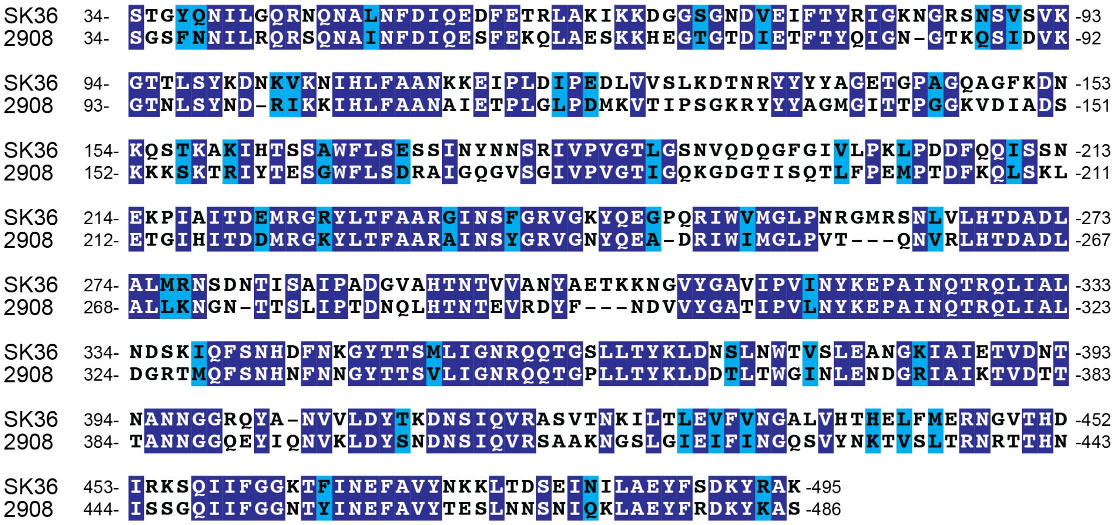

Fig. S4. Sequence alignment of the portions of PilC from SK36 and 2908 strains, which were structurally characterised in this study. Residues were shaded in dark blue (identical), light blue (conserved), or unshaded (different).

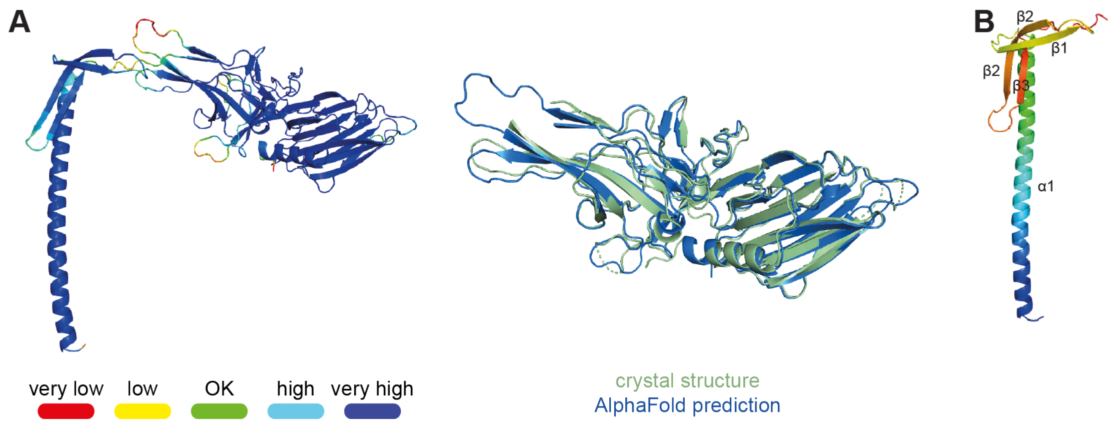

Fig. S5. AlphaFold model of PilC. A) Left, full-length model coloured following B-factor accuracy. Right, superposition of our crystal structure (green) with the corresponding portion of the AlphaFold (15) prediction (blue). The two structures show structural identity, superposing with an RMSD of 1.03 Å. **B**) Cartoon view of the of $PilC_{pilin}$ rainbow-coloured from blue (N-terminus) to red (C-terminus).

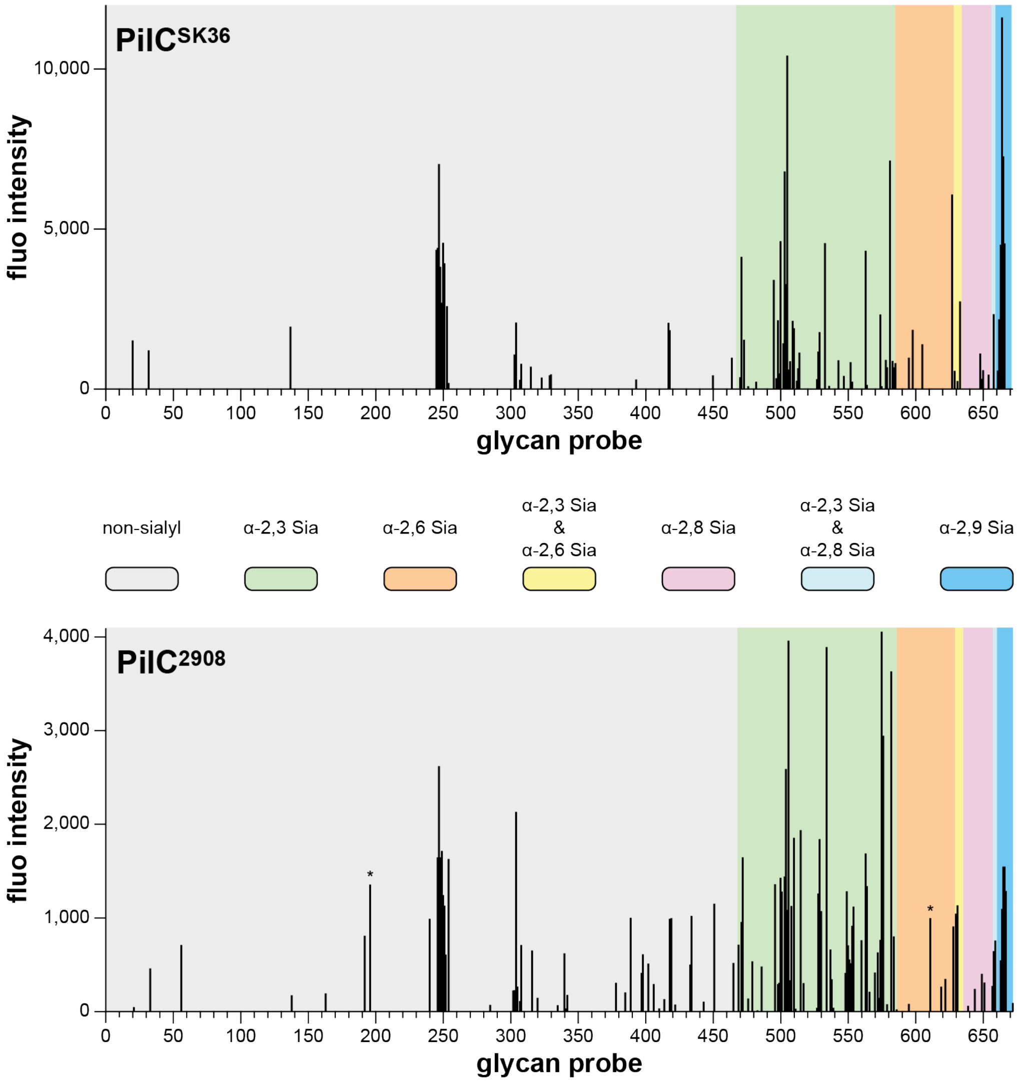

Fig. S6. Comparison of the glycan microarray analyses of the glycan-binding activities of PilC^{SK36} and PilC^{2908}. The results are the means of fluorescence intensities of duplicate spots, printed at 5 fmol/spot. In the glycan array the 672 lipid-linked probes are grouped according to sialyl linkages as indicated by the coloured panels. The full list of glycan probes, their sequences, and binding scores are given in Dataset S1. For easy comparison of the sialyl glycan probes bound by the two proteins, a "condensed matrix" focused on sialyl glycan probes has been added in Dataset S1. * Signals with large error bars due to artefacts on the array slides.

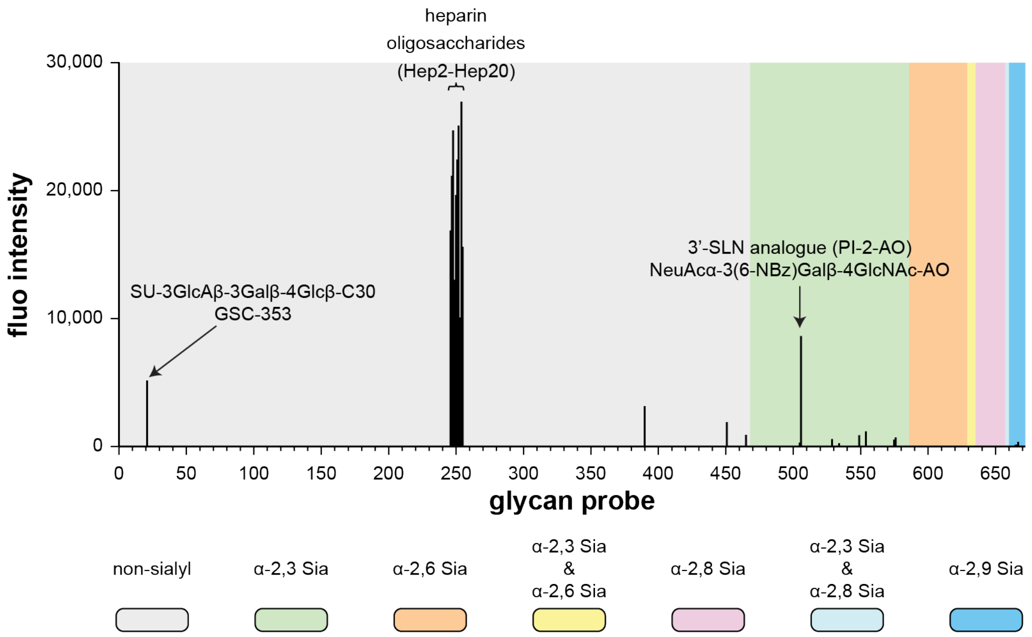

Fig. S7. Glycan microarray analysis of the glycan-binding activity of PilC$_{\Delta pilin}$. PilC$_{\Delta pilin}$ is the purified PilC protein without pilin module. The protein was analysed with and without precomplexation with detection antibodies, yielding similar results. The figure shows results with precomplexation. The results are the means of fluorescence intensities of duplicate spots, printed at 5 fmol/spot. In the glycan array the 672 lipid-linked probes are grouped according to sialyl linkages indicated by the coloured panels. The full list of glycan probes, their sequences, and binding scores are given in Dataset S1.

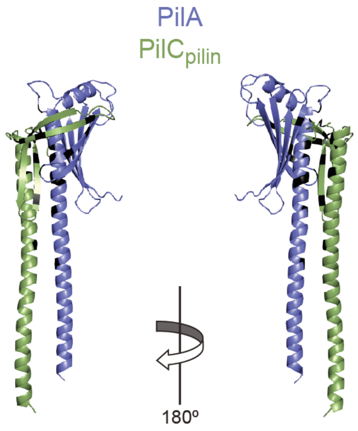

	PilC	dist. (Å)	PilA
**H bond**			
1	GLN_{34}	2.27	VAL_{24}
2	GLU_{49}	2.02	TYR_{42}
3	GLU_{49}	1.75	LYS_{41}
4	LYS_{56}	1.76	GLU_{147}
5	LYS_{56}	1.97	GLU_{147}
6	GLN_{71}	1.88	LYS_{85}
7	GLN_{71}	1.98	LYS_{85}
8	LYS_{83}	2.21	ASP_{57}
9	LYS_{95}	1.78	TYR_{35}
10	PHE_{99}	1.88	SER_{46}
11	ALA_{101}	2.36	GLN_{49}
12	GLU_{105}	2.00	GLN_{49}
13	THR_{106}	2.09	ILE_{144}
14	LEU_{108}	2.28	ILE_{144}
**Salt Bridge**			
1	GLU_{49}	2.64	LYS_{41}
2	LYS_{56}	2.60	GLU_{147}
3	LYS_{56}	2.80	GLU_{147}
4	ASP_{64}	3.86	LYS_{91}
5	GLU_{66}	2.77	HIS_{88}

Fig. S8. Interactions between PilA and PilC in AlphaFold model of the complex. Left, 180º view of the complex between PilA (blue) and the pilin module of PilC (green). Residues which stabilise the complex by forming a series of hydrogen bonds and salt bridges are highlighted in black. Right, the table lists the residues forming H bonds and/or salt bridges. Numbering is according to the mature pilins.

Table S1. Crystal structures data collection and refinement statistics.

**Protein** (PDB)	**PilA** (7O5Y)	**PilC** (7OA7)	**PilC^{SK36}** (7OA8)
Maximum resolution (Å)	1.77	1.45	1.60
Space group	$P4_32_12$	$P3_221$	$P2_12_12_1$
Unit cell parameters			
a, b, c (Å)	93.32, 93.32, 181.13	96.48, 96.48, 85.08	38.64, 96.03, 109.84
α, β, ɣ (°)	90, 90, 90	90, 90, 120	90, 90, 90
Number of observations	981,295	1,623,160	352,457
Number of unique observations	78,315	81,269	54,911
R_{merge} (%)	0.114	0.115	0.040
I/σI	12.1	15.4	17.2
CC ½	0.995	0.999	0.999
Resolution range used for refinement	37.34 – 1.77	83.56 – 1.45	48.01 – 1.60
Completeness (%)	99.6	99.9	99.9
R factor (%)	20.3	18.3	20.9
Free R factor (%)	23.4	20.1	23.9
Ramachandran favoured (%)	98.6	98.5	97.6
Ramachandran allowed (%)	1.4	1.5	2.4
Ramachandran outliers (%)	0	0	0
RMSD from ideal values			
bond length (Å)	0.007	0.007	0.005
bond angles (°)	1.1	0.994	0.730

Table S2. Strains and plasmids used in this study.

Name	Details*	Source
E. coli **strains**		
DH5α	used for cloning	
BL21(DE3)	used for protein expression/purification	
B834(DE3)	used for SeMet protein expression/purification	
**Plasmids (all pET-28b derivatives)**		
pET-28b	T7-based expression vector	Novagen
pET28-*pilA*	purify 6His-PilA$_{25\text{-}156}$ (**6His-PilA in the text**)	(3)
pET28-Strep-*pilA*	purify 6His-PilA$_{25\text{-}156}$ (**Strep-PilA**)	this study
pET28-Strep-*pilA*$_{A68S}$	purify Strep-PilA$_{25\text{-}156}$ with A68S mutation	this study
pET28-Strep-*pilA*$_{K85A}$	purify Strep-PilA$_{25\text{-}156}$ with K85A mutation	this study
pET28-Strep-*pilA*$_{T87A}$	purify Strep-PilA$_{25\text{-}156}$ with T87A mutation	this study
pET28-*pilB*	purify 6His-PilB$_{29\text{-}454}$ (**6His-PilB**)	(3)
pET28-Strep-*pilB*	purify Strep-PilB$_{29\text{-}454}$(**Strep-PilB**)	this study
pET28-*pilC*	purify 6His-PilC$_{25\text{-}477}$ (**6His-PilC**)	(3)
pET28-*pilC*^{SK36}	purify 6His-PilC$_{25\text{-}486}$ from SK36 (**6His-PilC^{SK36}**)	this study
pET28-*pilC*$_{\Delta pilin}$	purify 6His-PilC$_{112\text{-}477}$ (**6His-PilC$_{\Delta pilin}$**)	this study
pET28-*pilC*$_{T347A}$	purify 6His-PilC$_{112\text{-}477}$ with T347A mutation	this study
pET28-*pilC*$_{K349A}$	purify 6His-PilC$_{112\text{-}477}$ with K349A mutation	this study
pET28-*pilC*$_{T355A}$	purify 6His-PilC$_{112\text{-}477}$ with T355A mutation	this study
pET28-*pilC*$_{N359A}$	purify 6His-PilC$_{112\text{-}477}$ with N359A mutation	this study
pET28-*pilC*$_{E361A}$	purify 6His-PilC$_{112\text{-}477}$ with E361A mutation	this study
pET28-*pilC*$_{R365A}$	purify 6His-PilC$_{112\text{-}477}$ with R365A mutation	this study
pET28-*pilC*$_{G380S}$	purify 6His-PilC$_{112\text{-}477}$ with G380S mutation	this study
pET28-*pilC*$_{Q381A}$	purify 6His-PilC$_{112\text{-}477}$ with Q381A mutation	this study
pET28-*pilC*$_{Y383A}$	purify 6His-PilC$_{112\text{-}477}$ with Y383A mutation	this study
pET28-Strep-*pilC*	purify Strep-PilC$_{25\text{-}477}$ (**Strep-PilC**)	this study
pET28-*pilE1*	purify 6His-PilE1$_{28\text{-}139}$ (**6His-PilE1**)	(3)
pET28-Strep-*pilE1*	purify Strep-PilE1$_{28\text{-}139}$ (**Strep-PilE1**)	this study
pET28-*pilE2*	purify 6His-PilE2$_{28\text{-}132}$ (**6His-PilE2**)	(3)
pET28-Strep-*pilE2*	purify Strep-PilE2$_{28\text{-}132}$ (**Strep-PilE2**)	this study

*Numbering is according to the mature pilins. *pilB*, *pilC*, and *pilC*^{SK36} are codon-optimised synthetic genes.

Table S3. Primers used in this study.

Name	Sequence
pilA-pETF	ggg**ccatgg**atcatcatcatcatcatcaTGATACAGGGCAAAGCCAGAC
Strep-*pilA*-pETF	ggg**ccatgg**attggagccacccgcagttcgaaaagGATACAGGGCAAAGCCAGAC
pilA-pETR	ccc**gtcgac**TTACTTCTGTGCCGATCTCAA
pilA$_{A68S}$#1	GGCAATCCATCATCGGTTTATTCAGAGTTTGACGAGCGGGCCC
pilA$_{A68S}$#2	GGGCCCGCTCGTCAAACTCTGAATAAACCGATGATGGATTGCC
pilA$_{K85A}$#1	GATCCTTCAACAGAGCCGATTGCGTCAACCCATACCTTCAAAG
pilA$_{K85A}$#2	CTTTGAAGGTATGGGTTGACGCAATCGGCTCTGTTGAAGGATC
pilA$_{T87A}$#1	CAACAGAGCCGATTAAGTCAGCCCATACCTTCAAAGATGGC
pilA$_{T87A}$#2	GCCATCTTTGAAGGTATGGGCTGACTTAATCGGCTCTGTTG
pilB-pETF	ggg**ccatgg**atcatcatcatcatcatcaTAGCAGCCGTGAACTGATTGA
Strep-*pilB*-pETF	ggg**ccatgg**attggagccacccgcagttcgaaaagAGCAGCCGTGAACTGATTGA
pilB-pETR	ccc**ggatcc**TTACGGACCGCTAACAAACC
pilC-pETFbis	ggg**ccatgg**atcatcatcatcatcatcaTAGCGGCAGCTTTAATAACATTCTGCG
Strep-*pilC*-pETF	ggg**ccatgg**attggagccacccgcagttcgaaaagAGCGGCAGCTTTAATAACATTCTGCG
pilC-pETR	ccc**ggatcc**TTAGCTTGCTTTGTATTTATCGC
pilC$_{\Delta pilin}$	ggg**ccatgg**atcatcatcatcatcatcaTGATATGAAAGTGACCATTCCGAGCGGTAAACGTTATTAC
pilC$_{T347A}$#1	CAGCAGACAGGTCCGCTGCTGGCATATAAACTGGATGATACCC
pilC$_{T347A}$#2	GGGTATCATCCAGTTTATATGCCAGCAGCGGACCTGTCTGCTG
pilC$_{K349A}$#1	GGTCCGCTGCTGACATATGCACTGGATGATACCCTGAC
pilC$_{K349A}$#2	GTCAGGGTATCATCCAGTGCATATGTCAGCAGCGGACC
pilC$_{T355A}$#1	CTGGATGATACCCTGGCCTGGGGTATTAATC
pilC$_{T355A}$#2	GATTAATACCCCAGGCCAGGGTATCATCCAG
pilC$_{N359A}$#1	CCCTGACCTGGGGTATTGCTCTGGAAAATGATGGTC
pilC$_{N359A}$#2	GACCATCATTTTCCAGAGCAATACCCCAGGTCAGGG
pilC$_{E361A}$#1	CCTGGGGTATTAATCTGGCAAATGATGGTCGCATTGC
pilC$_{E361A}$#2	GCAATGCGACCATCATTTGCCAGATTAATACCCCAGG
pilC$_{R365A}$#1	GGTATTAATCTGGAAAATGATGGTGCCATTGCCATCAAAACCGTTGATAC
pilC$_{R365A}$#2	GTATCAACGGTTTTGATGGCAATGGCACCATCATTTTCCAGATTAATACC
pilC$_{G380S}$#1	CCACCACCGCAAATAATGGTTCTCAAGAATATATCCAGAACG
pilC$_{G380S}$#2	CGTTCTGGATATATTCTTGAGAACCATTATTTGCGGTGGTGG
pilC$_{Q381A}$#1	CACCGCAAATAATGGTGGTGCAGAATATATCCAGAACGTG
pilC$_{Q381A}$#2	CACGTTCTGGATATATTCTGCACCACCATTATTTGCGGTG
pilC$_{Y383A}$#1	CAAATAATGGTGGTCAAGAAGCTATCCAGAACGTGAAACTGG
pilC$_{Y383A}$#2	CCAGTTTCACGTTCTGGATAGCTTCTTGACCACCATTATTTG
pilC^{SK36}-pETF	ggg**ccatgg**atcatcatcatcatcatcatAGCACCGGTTATCAGAACATTCTG
pilC^{SK36}-pETR	ccc**ggatcc**TTATTTGGCACGGTATTTATCGC
pilE-pETF	gg**ccatgg**atcatcatcatcatcatcatcaAGATAACGCTCGTAAGAGCC
Strep-*pilE*-pETF	ggg**ccatgg**attggagccacccgcagttcgaaaagcaAGATAACGCTCGTAAGAGCC
pilE1-pETR	cc**ggatcc**TTAGTTTGAGTTTACACCATTAGCAGA
pilE2-pETR	cc**ggatcc**TTATTTTGAATTAGCACCAGCTTCG

pilB, *pilC*, and *pilC*^{SK36} are codon-optimised synthetic genes. Overhangs are in lower case. Restriction sites are in bold. Mismatches are in red.

Table S4. Glycan microarray specifications following the MIRAGE (minimum information required for a glycomics experiment) guidelines (17).

**1. Glycan binding sample**	
Description of sample	*Sample names*: PilC, $PilC_{\Delta pilin}$ and $PilC^{SK36}$. *Origin*: recombinant. All samples contained an N-terminal 6His tag. *Method of preparation*: see "Protein purification" section in the main text.
Sample modifications	Not relevant.
Assay protocol	Microarray analyses were performed essentially as described (7), for modifications of the protocol see "Glycan microarrays" section in the main text.
**2. Glycan library**	
Glycan description for defined glycans	A broad-spectrum screening microarray containing 672 sequence-defined oligosaccharide probes was used. The probe names, corresponding sequences, and IDs in the international glycan structure repository GlyTouCan (https://glytoucan.org/) are displayed in Dataset S1. These probes represent a subset of a recently generated large screening microarray containing around 900 glycan probes (in-house designation "Array sets 42-56", which will be published elsewhere). The NGL probes are from the collection assembled by the Glycosciences Laboratory (https://glycosciences.med.ic.ac.uk/glycanLibraryList.html).
Glycan description for undefined glycans	Not relevant.
Glycan modifications	No modification was carried out for natural glycolipids. NGLs, unless otherwise specified, were prepared by reducing oligosaccharides by reductive amination with the amino lipid, 1,2-dihexadecyl-*sn*-glycero-3-phosphoethanolamine (DHPE) (18). AO indicates NGLs prepared by reducing oligosaccharides by oxime ligation with an aminooxy functionalised DHPE (AOPE) (19). For full description of the definition of lipid moieties of the glycan probes see https://glycosciences.med.ic.ac.uk/docs/lipids.pdf.
**3. Printing surface**	
Description of surface	Nitrocellulose-coated glass microarray slides.
Manufacturer	16-pad UniSart 3D Microarray Slide from Sartorius (Goettingen, Germany).
Custom preparation of surface	Not relevant.
Non-covalent Immobilisation	The lipid-linked oligosaccharide probes, for arraying and non-covalent immobilisation on nitrocellulose-coated glass slides (7), were formulated as liposomes by adding carrier lipids, 1,2-dihexanoyl-*sn*-glycero-3-phosphocholine (DHPC) and cholesterol.
**4. Arrayer (Printer)**	
Description of arrayer	Nano-Plotter 2.1 from GeSiM (Radeberg, Germany).

Dispensing mechanism	Non-contact liquid delivery with four dispensing tips.
Glycan deposition	Approximately 0.33 nl was printed per spot. Lipid-linked glycan probes were printed at 2 and 5 fmol per spot in duplicate.
Printing conditions	The printing solutions were all aqueous based. Printing was performed at ambient temperature and relative humidity of 58 %. In addition fro the lipid-linked glycan probes, the "liposome" printing solutions contained 100 pmol/µl of DHPC and cholesterol as lipid carriers (both from Sigma). The concentrations of the lipid-linked glycan probes were 5 and 15 pmol/µl for the 2 and 5 fmol per spot levels, respectively. The printing solutions also contained Cy3 NHS ester (GE Healthcare) at 20 ng/ml (26 fmol/µl) as a marker to monitor the printing process.
**5. Glycan microarray with "map"**	
Array layout	Each array slide contained 16-pad subarrays. Each pad was set up for printing 64 probes maximum, each at 2 levels in duplicate (four spots for one probe in a row); 256 spots (16x16) in total in each pad. The 672 lipid-linked probes in the screening arrays were printed on multiple subarrays for parallel binding analyses.
Glycan identification and quality control	The quality control of the glycan microarrays was routinely carried out with a panel of (i) biotinylated plant lectins (Vector Laboratories) including *Ricinus communis* agglutinin I, *Aleuria aurantia* lectin, Concanavalin A, and wheat germ agglutinin, (ii) anti-carbohydrate antibodies, and (iii) commercial bacterial adhesins and toxins. Binding results will be shared via the GlyGen glycan array repository currently under development as part of the NIH-funded GlyGen iniatiative (https://www.glygen.org/), which has entered its final testing phase. In the meantime, datasets can be seen in the shared Google folder https://drive.google.com/drive/folders/1hMr-bWX4k3XxBd8FIB8cHbPkHBsxNzkj?usp=sharing. The sialylated glycan probes included in the present glycan array analyses have been extensively validated in previous studies with influenza viruses (20), and a number of viral adhesive proteins including VP1 proteins of polyomaviruses – simian virus 40 (21) and human JC polyomavirus (22) – and the fiber knob of human adenovirus 52 (23).
**6. Detector and data processing**	
Scanning hardware	GenePix 4300A from Molecular Devices (UK).
Scanner settings	*Scanning resolution*: 10 µm/pixel.<br>*Laser channel*: Red (scan wavelength 635 nm).<br>*PMT*: 350.<br>*Scan power*: various laser powers were used (indicated in Dataset S1) to record optimal scan images without spot saturation.
Image analysis software	GenePix® Pro 7 (Molecular Devices).
Data processing	The gpr files were entered into an in-house microarray database using software designed by Mark Stoll for data processing (http://www.beilstein-institut.de/en/publications/proceedings/glyco-2009). No particular normalisation method or statistical analysis was used for the results of the screening arrays.

7. Glycan microarray data presentation	
Data presentation	The microarray binding results are presented as histogram charts in Fig. 7A, Fig. S6 and Fig. S7. The results table with binding scores and relative binding intensities shown as "matrix" are in Dataset S1. This dataset also displays a focused matrix of the bound sialyl glycan probes with their sequences.
**8. Interpretation and conclusion from microarray data**	
Data interpretation	No software or algorithms were used to interpret processed data.
Conclusions	PilC, $PilC_{\Delta pilin}$ and $PilC^{SK36}$ bound to sialyl glycans and sulphated GAGs (mainly heparin) NGL probes in the screening array. $PilC_{\Delta pilin}$ showed much stronger binding to heparin NGL probes relative to sialyl probes. For the full-length proteins, PilC and $PilC^{SK36}$, very similar binding patterns were observed to a broad range of sialylated glycans, mainly sialyl α2-3-linked and α2-9-linked polysialic. Among the differences there is the probe NeuAcα-6GalNAc-AO bound strongly by $PilC^{SK36}$ and weakly by PilC.

Dataset S1. Results of the different glycan array experiments, with detailed list of probes on the array. This includes experiments performed with PilC, $PilC_{\Delta pilin}$ and $PilC^{SK36}$.

SI References

1. H. Inoue, H. Nojima, H. Okayama, High efficiency transformation of *Escherichia coli* with plasmids. *Gene* **96**, 23-28 (1990).

2. I. Gurung *et al.*, Functional analysis of an unusual type IV pilus in the Gram-positive *Streptococcus sanguinis*. *Mol. Microbiol.* **99**, 380-392 (2016).

3. J. L. Berry *et al.*, Global biochemical and structural analysis of the type IV pilus from the Gram-positive bacterium *Streptococcus sanguinis*. *J. Biol. Chem.* **294**, 6796-6808 (2019).

4. V. Y. Orekhov, V. A. Jaravine, Analysis of non-uniformly sampled spectra with multi-dimensional decomposition. *Prog. Nucl. Mag. Res. Spect.* **59**, 271-292 (2011).

5. F. Delaglio *et al.*, NMRPipe: a multidimensional spectral processing system based on UNIX pipes. *J. Biomol. NMR* **6**, 277-293 (1995).

6. W. Lee, M. Tonelli, J. L. Markley, NMRFAM-SPARKY: enhanced software for biomolecular NMR spectroscopy. *Bioinformatics* **31**, 1325-1327 (2015).

7. Y. Liu *et al.*, Neoglycolipid-based oligosaccharide microarray system: preparation of NGLs and their noncovalent immobilization on nitrocellulose-coated glass slides for microarray analyses. *Meth. Mol. Biol.* **808**, 117-136 (2012).

8. U. Neu *et al.*, A structure-guided mutation in the major capsid protein retargets BK polyomavirus. *PLoS Pathog.* **9**, e1003688 (2013).

9. C. Marck, 'DNA Strider': a 'C' program for the fast analysis of DNA and protein sequences on the Apple Macintosh family of computers. *Nucleic Acids Res.* **16**, 1829-1836 (1988).

10. P. Jones *et al.*, InterProScan 5: genome-scale protein function classification. *Bioinformatics* **30**, 1236-1240 (2014).

11. L. Holm, L. M. Laakso, Dali server update. *Nucleic Acids Res.* **44**, W351-355 (2016).

12. K. Sumathi, P. Ananthalakshmi, M. N. Roshan, K. Sekar, 3dSS: 3D structural superposition. *Nucleic Acids Res.* **34**, W128-W132 (2006).

13. R. B. Russell, G. J. Barton, Multiple protein sequence alignment from tertiary structure comparison: assignment of global and residue confidence levels. *Proteins* **14**, 309-323 (1992).

14. E. Krissinel, K. Henrick, Inference of macromolecular assemblies from crystalline state. *J. Mol. Biol.* **372**, 774-797 (2007).

15. J. Jumper *et al.*, Highly accurate protein structure prediction with AlphaFold. *Nature* **596**, 583-589 (2021).

16. R. Evans *et al.*, Protein complex prediction with AlphaFold-Multimer. *BioRxiv* https://doi.org/10.1101/2021.10.04.463034 (2022).

17. Y. Liu *et al.*, The minimum information required for a glycomics experiment (MIRAGE) project: improving the standards for reporting glycan microarray-based data. *Glycobiology* **27**, 280-284 (2017).

18. W. Chai, M. S. Stoll, C. Galustian, A. M. Lawson, T. Feizi, Neoglycolipid technology: deciphering information content of glycome. *Meth. Enzymol.* **362**, 160-195 (2003).

19. Y. Liu *et al.*, Neoglycolipid probes prepared via oxime ligation for microarray analysis of oligosaccharide-protein interactions. *Chem. Biol.* **14**, 847-859 (2007).

20. R. A. Childs *et al.*, Receptor-binding specificity of pandemic influenza A (H1N1) 2009 virus determined by carbohydrate microarray. *Nat Biotechnol* **27**, 797-799 (2009).

21. H. Ewers *et al.*, GM1 structure determines SV40-induced membrane invagination and infection. *Nat Cell Biol* **12**, 11-18; sup pp 11-12 (2010).

22. U. Neu *et al.*, Structure-function analysis of the human JC polyomavirus establishes the LSTc pentasaccharide as a functional receptor motif. *Cell Host Microbe* **8**, 309-319 (2010).

23. A. Lenman *et al.*, Polysialic acid is a cellular receptor for human adenovirus 52. *Proc. Natl. Acad. Sci. USA* **115**, E4264-E4273 (2018).
